# Synthesis of the Abiraterone Derivatives 5α‐ and 5β‐Δ^1^‐Abiraterone and Structural Determination of an Unknown Metabolite in Human Serum Derived from Oral Abiraterone Acetate

**DOI:** 10.1002/cbic.202500675

**Published:** 2025-11-18

**Authors:** Noboru Hayama, Shizuyo Horiyama, Ryoma Okamoto, Yukina Nishi, Manae Yada, Ryuji Higashida, Jun Haginaka, Yoshihide Usami

**Affiliations:** ^1^ Faculty of Pharmacy Osaka Medical and Pharmaceutical University 4‐20‐1 Nasahara Takatsuki Osaka 569–1094 Japan; ^2^ School of Pharmacy and Pharmaceutical Sciences Mukogawa Women's University 11–68 Koshien Kyuban‐cho Nishinomiya 663–8179 Japan; ^3^ Institute for Biosciences Mukogawa Women's University 11–68 Koshien Kyuban‐cho Nishinomiya 663–8179 Japan

**Keywords:** abiraterone, metabolite, olefination, prostate cancer, steroid

## Abstract

Abiraterone (Abi) acetate, an anticancer drug, produces metabolites in the serum of patients with prostate cancer. Three unknown metabolites of Abi are previously discovered and the structures of two of these compounds are determined by comparing their LC‐based retention times with those of synthesized compounds. However, the structure of the third metabolite is not identified. Herein, two new Abi derivatives, 5α‐Δ^1^‐abiraterone (5α‐D1A) and 5β‐Δ^1^‐abiraterone (5β‐D1A), with a molecular weight of 348, are successfully synthesized as candidates for the third unknown metabolite. 5β‐D1A is confirmed as this third metabolite based on its retention time in the extracted ion chromatogram. The metabolic pathway may have formed 5β‐D1A rather than 5α‐D1A because of the higher activity of steroid 5β‐reductase compared with that of 5α‐reductase. These findings are expected to lead to the discovery of new metabolic pathways.

## Graphical abstract

Abiraterone (Abi) acetate, a prostate cancer treatment, is orally administered and hydrolyzed, and multiple metabolites are produced in serum by various enzymes. Two novel Abi derivatives, 5α‐ and 5β‐Δ^1^‐abiraterone, are synthesized. Then, an unknown metabolite in serum is identified to be 5β‐Δ^1^‐abiraterone based on its extracted ion chromatogram retention time.

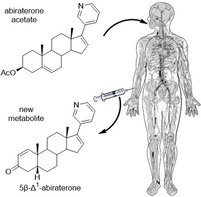

## 1 Introduction

Prostate cancer statistics for 2020 estimated over 1,400,000 new cases and over 370,000 deaths worldwide.^[^ ^1^ ^]^ The progression of prostate cancer is associated with androgen receptors activated by testosterone, androstenedione, and dehydroepiandrosterone (DHEA), which are synthesized in the testes or adrenal glands.^[^ ^2^ ^]^ The standard treatment for patients with advanced prostate cancer is androgen deprivation therapy via surgical or medical castration.^[^ ^3^ ^]^ However, during the long‐term treatment of patients with advanced cancer, androgen receptor signaling is reactivated, leading to castration‐resistant prostate cancer (CRPC).^[^ ^4^ ^]^ The development of drugs to block androgen receptors during CRPC treatment has led to the discovery of enzalutamide, which is more potent than previously discovered androgen receptor antagonists such as bicalutamide.^[^ ^5^ ^]^

Strategies targeting cytochrome P450 c17 (CYP17), a crucial enzyme for androgen synthesis, have emerged as alternative treatment methods for prostate cancer. CYP17 catalyzes oxidation at the C‐17 position of pregnenolone and progesterone, which are key precursors in androgen biosynthesis.^[^ ^6^ ^]^ Abiraterone (Abi), the structure of which comprises a pyridine group attached to C‐17 of DHEA, was previously discovered as a potent and selective inhibitor of CYP17.^[^ ^7^ ^]^ Abi acetate is clinically used as an oral prodrug and hydrolyzes into Abi in vivo.^[^ ^8^ ^]^ Abi is converted into several metabolites exhibiting various activities by enzymes such as 3β‐hydroxysteroid dehydrogenase (3β‐HSD), 5α‐reductase, and 5β‐reductase.^[^ ^9^ ^]^ Interestingly, Δ^4^‐abiraterone (D4A), an oxidized and double bond‐isomerized metabolite of Abi produced by 3β‐HSD/Δ^5^‐Δ^4^‐isomerase, exhibits higher antitumor activity than the parent compound.^[^ ^10^ ^]^ 3‐Keto‐5α‐Abi, an α‐face hydrogenated metabolite of D4A produced by the action of α‐reductase, is an androgen receptor agonist.

**Scheme 1:**
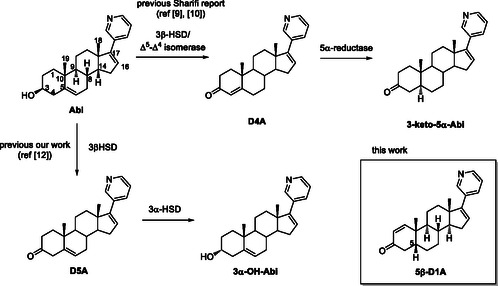
Plausible metabolic pathways of Abi.

The development of new analytical methods to monitor Abi and its metabolites after Abi acetate administration is necessary. We previously developed a solid‐phase extraction (SPE) approach coupling silica cartridges and an liquid chromatography‐electrospray ionization‐time of flight/mass spectrometry (LC‐ESI‐TOF/MS) method to comprehensively detect Abi and its metabolites in human serum after Abi acetate administration.^[^ ^11^ ^]^ Using this strategy, we found three unknown metabolites and identified two of them as 3α‐OH‐Abi and Δ^5^‐Abi (D5A) (**Scheme** **1**).^[^ ^12^ ^]^ The putative biosynthetic pathway showed that 3α‐hydroxysteroid dehydrogenase participated in the metabolic pathway of Abi in vivo. However, the structure of the third metabolite, the [M + H]^+^ of which showed a signal at *m/z* 348.2322 was not identified. Hence, in this study, we designed two plausible structural isomers of this metabolite: 5α‐Δ^1^‐abiraterone (5α‐D1A) and 5β‐Δ^1^‐abiraterone (5β‐D1A). We report the synthesis of these metabolites and identify the remaining unknown Abi metabolite by comparing its extracted ion chromatogram (EIC) retention time with those of the synthesized compounds using a column capable of separating steroid isomers. Almost no Δ^1^ compounds formed through human steroid metabolic pathways have been reported.^[^ ^13^ ^]^ Clarifying the structure of Δ^1^‐abiraterone (D1A) could lead to the discovery of new metabolic pathways, which would be significant.

## 2 Results and Discussion

**Figure 1:**
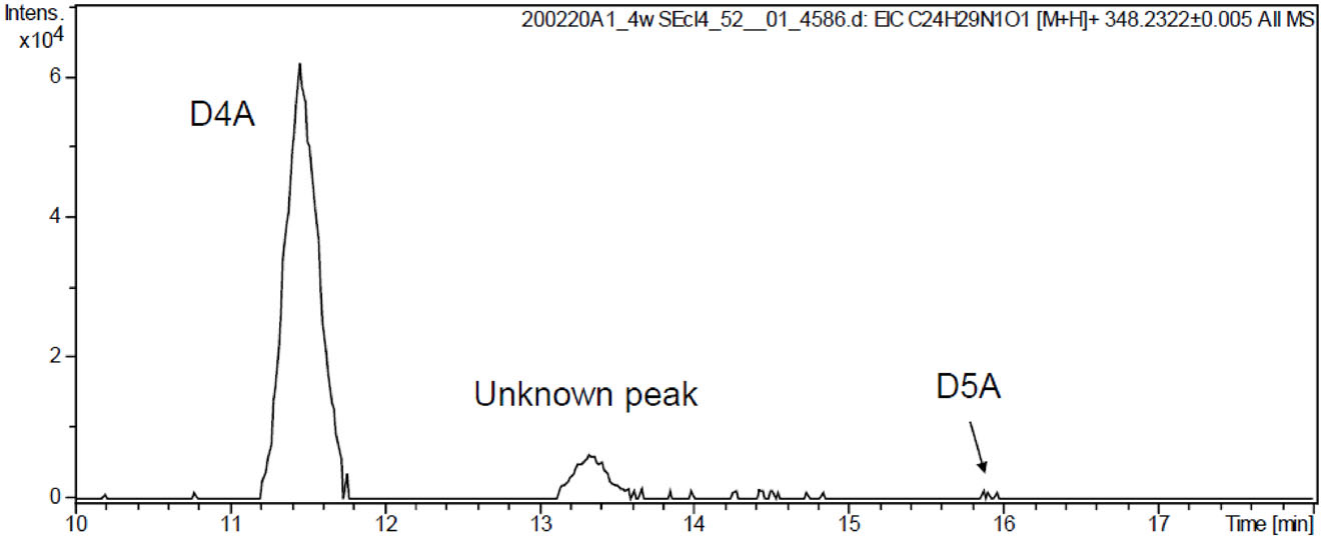
EIC of Abi and its metabolites recorded at *m/z* 348.2322 after Abi acetate administration.

The mass value of the unknown metabolite (unknown peak in **Figure** **1**) was identical to that of D4A, with the [M + H]^+^ ion revealing a mass of *m/z* 348.2322 ± 0.005 on the EIC. The retention time of the metabolite on the C18 column was between those of D4A and D5A. Therefore, D4A may have multiple isomers, depending on the position of the double bond. We hypothesized that this compound was a ketone conjugated with a Δ^1^‐olefin instead of the Δ^4^‐olefin in D4A and named it D1A. To identify this trace substance, we synthesized a standard substance for comparison.

**Scheme 2:**
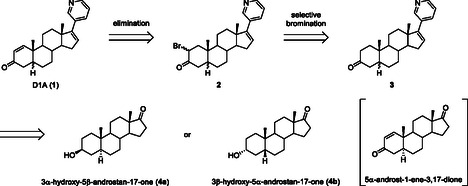
Retrosynthesis of D1A.

As D1A has a chiral center at C‐5, the third unknown metabolite should be one of two isomers: 5α‐D1A (**1a**), with a hydrogen atom on the α‐face, or 5β‐D1A (**1b**), with a hydrogen atom on the β‐face. The structure seemed impossible to determine because the pure compound had not been isolated from the obtained serum sample. Therefore, we synthesized both possible isomers, and their retrosynthetic routes are shown in **Scheme** **2** **.** The starting materials were selected to be 3β‐hydroxy‐5α‐androstan‐17‐one (**4a**) or 3α‐hydroxy‐5β‐androstan‐17‐one (**4b**) since both of which are commercially available and have the desired stereochemistry at C‐5. Both syntheses required the selective formation of the double bond between C‐1 and C‐2 and the introduction of a pyridine ring. The double bond between C‐1 and C‐2 could be formed by the selective bromination of the C‐2 position adjacent to the C‐3 carbonyl group and a subsequent elimination reaction.^[^ ^14^ ^,^ ^15^ ^]^ This double bond was formed in a latter step during the retrosynthesis because our earlier attempt at triflation, which is necessary to introduce the pyridine ring by Suzuki–Miyaura coupling, of the known 5α‐androst‐1‐ene‐3,17‐dione^[^ ^16^ ^]^ was unsuccessful. After protecting the hydroxyl group at C‐3 in starting **4a** or **4b**, the pyridine ring was introduced by Suzuki–Miyaura coupling. Deprotection followed by oxidation of the hydroxy group at C‐3 would afford Abi derivative **3**. The desired D1As **1** was then synthesized from **3**s via the brominated **2**s.

**Scheme 3:**
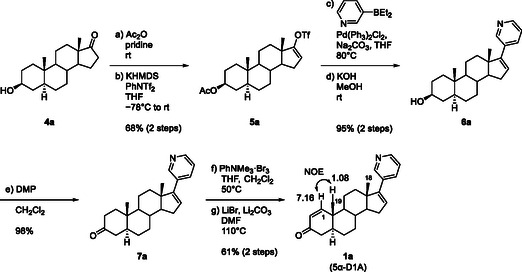
Synthesis of 5α‐D1A (**1a**).

**Scheme** **3** summarizes the synthesis of **1a** from **4a**. 3β‐OH‐5α‐Abi (**6a**) was prepared from **4a** as reported in the literature.^[^ ^9^ ^]^ The hydroxyl group at C‐3 in **4a** was protected and then converted to enol triflate **5a**. Subsequently, the obtained triflate was subjected to Suzuki coupling to introduce a pyridine ring. Deprotection of the acetyl group afforded **6a**. Oxidation of **6a** was performed with Dess–Martin periodinane to afford the desired product, 3‐keto‐5α‐Abi (**7a**), in 98% yield. The C‐1=C‐2 double bond was introduced by dissolving **7a** in CH_2_Cl_2_, adding a solution of trimethylaniline bromide (PhNMe_3_·Br) in THF, and heating at 50 °C to obtain a complex mixture containing brominated species. The mixture was heated without purification with Li_2_CO_3_ and LiBr at 110 °C to give **1a** in 61% yield.^[^ ^14^ ^]^ The structure of **1a** was confirmed by nuclear magnetic resonance (NMR) analysis. The two doublet olefinic protons in the ^1^H‐NMR spectrum of **1a** suggested the presence of a C‐1=C‐2 double bond. Observation of a nuclear overhauser effect spectroscopy (NOESY) cross peak between H‐1 and 19‐CH_3_ supported the position of the double bond. Another methyl group at C‐18 appeared at a higher field in the 1D ^1^H‐NMR spectrum owing to the effect of the pyridine ring. Observation of a molecular ion peak at *m/z* 348.2321 in the mass spectrum also supported the structure **1a**.

**Scheme 4:**
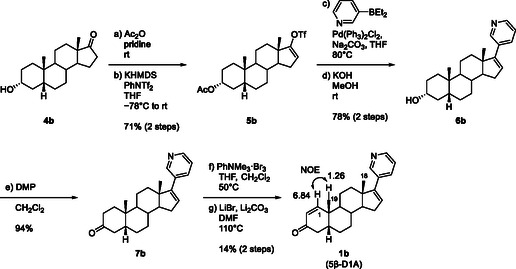
Synthesis of 5β‐D1A (**1b**).

The synthesis of **1b** from commercially available **4b** was carried out using procedures similar to those for **1a** (**Scheme** **4**). 3α‐OH‐5β‐Abi (**6b**) synthesized from **4b** was converted to 3‐keto‐5β‐Abi (**7b**) by Dess–Martin oxidation. Although the previous steps showed similar reactivity to **7a**, bromination at the *cis*‐fusion site of the AB ring steroid skeleton resulted in a more complex mixture. Nevertheless, the desired reaction proceeded satisfactorily, and the subsequent E2 elimination reaction gave the desired olefin **1b** in 14% yield, which was sufficient for analysis. **1b** was analyzed by NMR and MS, similar to **1a**, to confirm its structure. Compared with that in **1a**, the *cis*‐fusion AB ring of the steroid in **1b** was significantly altered, with a downfield shift in the signal of H‐1 and an upfield shift in the signal of the 19‐CH_3_ methyl group.

**Figure 2:**
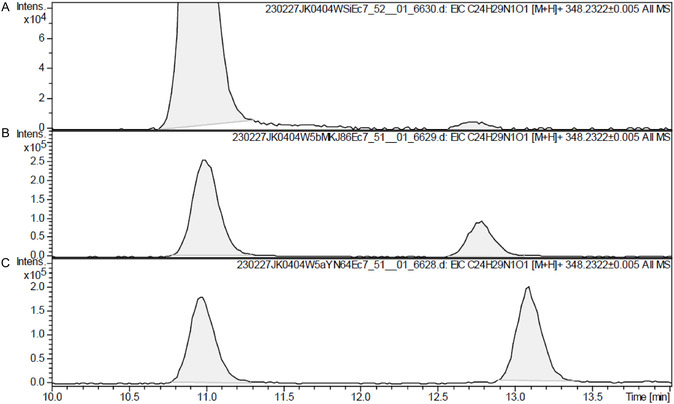
EICs of serum samples A) after Abi acetate administration, B) spiked with synthesized 5β‐D1A, and C) spiked with synthesized 5α‐D1A. The EICs were recorded at *m/z* 348.2322.

When the synthesized **1a** and **1b** were analyzed individually by LC‐ESI‐TOF/MS, their retention times were slightly different from those of the serum samples. This is probably because unknown compounds in the serum matrix (including D4A) were compared with **4a** and **4b**, which were dissolved in organic solvents. This resulted in a difference in retention time compared with that of a single compound. Serum samples obtained from patients that had received oral Abi acetate were added with the synthesized **1a** or **1b** and analyzed by LC‐ESI‐TOF/MS (**Figure** **2**). The retention time of **1a** was longer than that of the unknown metabolite, whereas that of **1b** was completely consistent with it. Therefore, the new unknown metabolite was identified as 5β‐D1A.

The conclusion that the unknown metabolite was 5β‐D1A instead of 5α‐D1A is reasonable because the patients were of East Asian descent. The activity of steroid 5α‐reductase, an enzyme that reduces the C‐5 position of the steroid skeleton on the α‐face, is relatively low in East Asians, and the enzyme aldo‐keto reductase family 1 member D1 is presumed to reduce the C‐5 position on the β‐face.^[^ ^17^ ^]^ Thus, we hypothesized that we would detect a metabolite in which the C‐5 position of the steroid was selectively reduced to the β‐position. However, the metabolic pathway to 5β‐D1A has not yet been elucidated, and further exploration is required in future work.

Similar to the previously reported novel metabolites 3α‐OH‐Abi and D5A, the structure of 5β‐D1A was identified. The combination of silica‐cartridge‐based SPE and LC‐ESI‐TOF/MS enabled the more detailed detection of metabolites present in the serum of patients who had been orally administered Abi acetate. Further research aiming to clarify the influence of these three metabolites on the androgen receptor will help establish a treatment for prostate cancer. Therefore, as we have not yet been able to synthesize the minimum amount required for analysis, the selective olefin formation reaction should be further improved to obtain sufficient samples for further insights.

## 3 Conclusion

Two isomers of D4A, one of which was an oxidized metabolite of Abi, were synthesized, and each isomer possessed a double bond at C‐1 instead of C‐4. This synthesis elucidated that the third unidentified blood metabolite formed following the oral administration of Abi acetate was 5β‐D1A, which has a unique structure comprising a *cis*‐fuzed ring with double bonds at C‐1 and C‐2 of the steroid skeleton. The reason why 5β‐D1A was formed as a metabolite rather than 5α‐D1A may have been the higher activity of steroid 5β‐reductase compared with that of 5α‐reductase. Ultimately, the pharmacological activity of new metabolites must be clarified; to that end, the combination of silica‐cartridge‐based SPE and LC‐ESI‐TOF/MS enables the detection of unknown metabolites in more detail than traditional methods.

## 4 Experimental Section

4.1

4.1.1

General information and the synthetic procedures for compounds **5a**–**7a** and **5b**–**7b** are available in the Supporting Information.

##### Synthesis of 5α‐D1A

**17‐(3‐Pyridyl)‐5α‐androsta‐1,16‐dien‐3‐one (1a, 5α‐D1A)**: To a solution of **7a** (105 mg, 0.30 mmol) in 5 mL of CH_2_Cl_2_ was added a solution of PhNMe_3_·Br_3_ (230 mg, 0.45 mmol) in THF (50 mL) at 0 °C. The reaction mixture was stirred at 50 °C for 3 h, cooled, and added with H_2_O. The resulting mixture was extracted twice with EtOAc, dried over Na_2_SO_4_, and concentrated in vacuo. The obtained crude residue was used for the next step without further purification. To a solution of the mixture in DMF (10 mL) were added Li_2_CO_3_ (44 mg, 0.60 mmol) and LiBr (78 mg, 0.90 mmol) at room temperature. The reaction mixture was stirred at 110 °C for 3 h, cooled, and added with H_2_O. The resulting mixture was extracted twice with EtOAc, dried over Na_2_SO_4_, and concentrated in vacuo. The residue was purified by column chromatography on silica gel (*n*‐hexane:ethyl acetate = 4:1–1:1) to afford **1a** (63 mg, 61%) as a white solid. ^1^H NMR (400 MHz, CDCl_3_) *δ* 8.65–8.60 (m, 1H), 8.51–8.44 (m, 1H), 7.65 (ddd, *J* = 7.9, 1.7, 1.7 Hz, 1H), 7.23 (dd, *J* = 7.9, 4.8 Hz, 1H), 7.16 (d, *J* = 10.3 Hz, 1H), 5.99 (dd, *J* = 3.1, 1.7 Hz, 1H), 5.87 (d, *J* = 10.3 Hz, 1H), 2.40 (dd, *J* = 17.6, 14.2 Hz, 1H), 2.31–2.21 (m, 2H), 2.13–1.43 (m, 11H), 1.22–1.00 (m, 9H); ^13^C NMR (100 MHz, CDCl_3_) *δ* 200.1, 158.1, 151.5, 147.9, 147.8, 133.6, 132.8, 129.2, 127.5, 123.1, 57.2, 50.2, 47.5, 44.4, 41.0, 39.2, 35.2, 34.2, 31.6, 31.1, 27.5, 21.1, 16.8, 13.1; IR (ATR): 2931, 1675, 798, 712 cm^–1^; mp 171–173 °C (EtOAc); [*α*]_D_^20^ + 110.3 (c 1.00, CHCl_3_); HRMS (ESI) *m/z* [M + H]^+^ calcd for C_24_H_29_NO 348.2322; found 348.2321.

##### Synthesis of 5β‐D1A

**17‐(3‐Pyridyl)‐5β‐androsta‐1,16‐dien‐3‐one (1b, 5α‐D1A)**: The experimental procedure for the synthesis of **1b** from **7b** (105 mg, 0.30 mmol) was identical to that for **1a**. Purification by column chromatography on silica gel (*n*‐hexane:ethyl acetate = 4:1–1:1) afforded **1b** (14 mg, 14%) as a white amorphous solid. ^1^H NMR (400 MHz, CDCl_3_) *δ* 8.64–8.56 (m, 1H), 8.51–8.42 (m, 1H), 7.63 (ddd, *J* = 7.9, 1.9, 1.9 Hz, 1H), 7.22 (dd, *J* = 7.9, 4.8 Hz, 1H), 6.84 (d, *J* = 10.3 Hz, 1H), 5.98 (dd, *J* = 3.3, 1.9 Hz, 1H), 5.92 (d, *J* = 10.3 Hz, 1H), 2.80 (dd, *J* = 18.1, 15.6 Hz, 1H), 2.28 (ddd, *J* = 15.6, 6.4, 3.3 Hz, 1H), 2.19–1.39 (m, 13H), 1.31–1.21 (4H), 1.05 (s, 3H); ^13^C NMR (100 MHz, CDCl_3_) *δ* 200.7, 161.2, 151.6, 147.9, 147.8, 133.7, 132.7, 129.0, 127.0, 123.0, 56.7, 47.5, 46.5, 41.0, 39.0, 38.7, 35.2, 33.8, 31.7, 26.3, 25.9, 22.2, 20.8, 16.7; IR (ATR): 2929, 1677, 798, 713 cm^–1^; [*α*]_D_^20^ + 149.7 (c 0.99, CHCl_3_); HRMS (ESI) *m/z* [M + H]^+^ calcd for C_24_H_29_NO 348.2322; found 348.2324.

##### Spiking Serum with 5α‐ and 5β‐D1A

The serum was obtained from patients undergoing a phase‐II clinical trial investigating the combination therapy of Abi and dutasteride for CRPC (approval number H29‐015−2).^[^ ^18^ ^]^ Written informed consent was obtained from all patients. The trial was reviewed and approved by the Ethics Review Committee for Medical Research Involving Human Subjects at Yamaguchi University School of Medicine and Yamaguchi University Hospital. After 4 weeks of treatment with Abi acetate (1000 mg daily) and prednisolone (10 mg daily), prior to initiating the dutasteride treatment, blood samples were collected from patients 3 ± 1 h after the final Abi acetate dose. The serum was pretreated using SPE.^[^ ^11^ ^]^ Then, an equal amount of a 10 ng mL^−^^1^ solution of synthesized 5α‐D1A or 5β‐D1A was added, and LC/MS analysis was performed by recording the EIC at *m/z* 348.2322.^[11]^

## Supporting Information

General information; the synthetic procedures for compounds **5a**–**7a** and **5b**–**7b**; and the ^1^H‐, ^13^C‐, and ^19^F‐NMR spectra of all compounds, and the COSY and NOESY spectra of **1a** and **b** are available in Supporting Information.

## Conflict of Interest

The authors declare no conflict of interest.

## Author Contributions

**Noboru Hayama**: conceptualization (lead); data curation (lead); formal analysis (lead); investigation (lead); methodology (lead); project administration (lead); resources (lead); supervision (lead); validation (lead); visualization (lead); writing—original draft (lead); writing—review and editing (lead). **Shizuyo Horiyama**: data curation (equal); formal analysis (equal); funding acquisition (lead); investigation (equal); methodology (equal); project administration (equal); resources (lead); validation (equal); visualization (equal); writing—review and editing (equal). **Ryoma Okamoto**: formal analysis (equal); supervision (supporting). **Yukina Nishi**: formal analysis (supporting). **Manae Yada**: formal analysis (supporting). **Ryuji Higashida**: formal analysis (supporting). **Jun Haginaka**: conceptualization (equal); supervision (equal); writing—review and editing (equal). **Yoshihide Usami**: conceptualization (equal); resources (equal); supervision (equal); writing—review and editing (equal).

## Supporting information

Supplementary Material

## Data Availability

The data that support the findings of this study are available in the supplementary material of this article.
