# Supplementary material for: Synthesis of the Abiraterone Derivatives 5α‐ and 5β‐Δ1‐Abiraterone and Structural Determination of an Unknown Metabolite in Human Serum Derived from Oral Abiraterone Acetate
Source: Chembiochem. 2025 Nov 18;26(24):e202500675. doi: 10.1002/cbic.202500675 (PMC12703417; doi:10.1002/cbic.202500675)
Supplement: Supplementary file 1 — Supplementary Material [file CBIC-26-e202500675-s001.pdf]

Supporting Information

for

Synthesis of the Abiraterone Derivatives 5α- and 5β-Δ^1-Abiraterone and Structural Determination of an Unknown Metabolite in Human Serum Derived from Oral Abiraterone Acetate

Noboru Hayama,*[a] Shizuyo Horiyama, *[b] Ryoma Okamoto, [a] Yukina Nishi, [a] Manae Yada, [a] Ryuji Higashida, [a] Jun Haginaka[c] and Yoshihide Usami[a]

[a] Faculty of Pharmacy, Osaka Medical and Pharmaceutical University,
4-20-1 Nasahara, Takatsuki, Osaka 569-1094, Japan
E-mail: noboru.hayama@ompu.ac.jp
[b] School of Pharmacy and Pharmaceutical Sciences, Mukogawa Women's University,
11-68, Koshien Kyuban-cho, Nishinomiya 663-8179, Japan
E-mail: horiyama@mukogawa-u.ac.jp
[c] Institute for Biosciences, Mukogawa Women's University,
11-68, Koshien Kyuban-cho, Nishinomiya 663-8179, Japan

TABLE OF CONTENTS

1. General Information..S2

2. Synthetic Procedure..S3

3. Copies of NMR charts..S5

1. General Information

All nonaqueous reactions were performed under an Ar atmosphere in dried glassware unless otherwise noted. Moreover, unless otherwise noted, all materials and solvents were purchased from Tokyo Kasei Co., Aldrich Inc., and other commercial suppliers and used without purification. Column chromatography was performed on Fuji Silysia silica gel 127 (100−270 mesh). Reactions and chromatography fractions were analyzed using precoated silica gel plates (Silica gel 70 F254-Wako). ^{1}H NMR (400 MHz) spectra were recorded using a JNM-ECZL400S spectrometer, and chemical shifts are reported in δ (ppm) relative to TMS (in $CDCl_3$) as the internal standard. Unless otherwise noted, ^{13}C NMR (100 MHz) spectra were also recorded using a JNM-ECZL400S spectrometer and referenced to the residual $CHCl_3$ signals. ^{1}H NMR multiplicities are reported as follows: br = broad; m = multiplet; s = singlet; d = doublet. IR spectra were measured on a Shimadzu IRAffinity-1S instrument. All melting points were measured on Yanagimoto micro melting point apparatus and are reported uncorrected. Optical rotations were recorded on a JASCO P-2300 polarimeter with a path length of 10 cm; concentrations are quoted in grams per 100 mL. $[\alpha]_D$ values are reported in 10^{-1} deg cm^2g^{-1}. High-resolution mass spectra were obtained in ESI positive ion mode on an Impact II TOF/MS system (Bruker Daltonics, Bremen, Germany) equipped with a Nexera SFC/SFE-HPLC system (Shimadzu, Kyoto, Japan)

.

2. Synthetic Procedure

3β-Acetoxy-5α-androsta-16-ene-17-yl-trifluoromethanesulfonate (**5a**): To a solution of 3β-hydroxy-5α-androstan-17-one (**4a**, 871 mg, 3.0 mmol) in pyridine (10 mL) was added acetic anhydride (6 mL, 60 mmol) at room temperature. The reaction mixture was stirred at room temperature for 6 h, cooled, and added with 1 M aqueous HCl. The resulting mixture was extracted with EtOAc. The organic layer was washed with saturated aqueous $NaHCO_3$, dried over Na_2SO_4, and concentrated in vacuo. The obtained crude residue was used for the next step without further purification. To a solution of the mixture in THF (30 mL) was added potassium bis(trimethylsilyl)amide (1.0 M THF solution, 3.3 mL, 3.3 mmol) at –78 °C. The reaction solution was stirred for 1 h at –78 °C and then added with a solution of *N*-phenyl-bis(trifluoromethanesulfonimide) (1.29 g, 3.6 mmol) in THF (20 mL) at –78 °C. The resultant solution was stirred for 1 h at –78 °C, warmed to room temperature for an additional 1 h, and added with saturated aqueous NH_4Cl. The resulting mixture was extracted twice with EtOAc, dried over Na_2SO_4, and concentrated in vacuo. The residue was purified by column chromatography on silica gel (*n*-hexane:ethyl acetate = 20:1) to afford **5a** (944 mg, 68%) as a colorless oil. ^{1}H NMR (400 MHz, $CDCl_3$) δ 5.56 (dd, *J* = 3.3, 1.7 Hz, 1H), 4.74–4.63 (m, 1H), 2.20 (ddd, *J* = 14.9, 5.7, 3.5 Hz, 1H), 2.02 (s, 3H), 2.01–1.93 (m, 1H), 1.84–1.78 (m, 1H), 1.76–1.15 (m, 10H), 1.08–0.89 (m, 9H), 0.85 (s, 3H), 0.83–0.75 (m, 1H); ^{13}C NMR (100 MHz, $CDCl_3$) δ 170.7, 159.2, 118.5 (q, $^1J_{C\text{-}F}$ = 320 Hz), 114.4, 73.4, 54.5, 54.1, 44.79, 44.77, 36.4, 35.7, 33.9, 33.4, 32.6, 30.7, 28.5, 28.2, 27.3, 21.4, 20.4, 15.2, 12.1; ^{19}F NMR (376 MHz, $CDCl_3$) δ –73.5; IR (ATR): 2941, 1731, 1421, 1209, 757 cm^{-1}; $[\alpha]_D^{20}$ +6.6 (c 1.00, CHCl3); HRMS (EI) *m/z* $[M]^+$ calcd for $C_{22}H_{31}F_3O_5S$ 464.1844; found 464.1844.

17-(3-Pyridyl)-5α-androsta-16-ene-3β-ol (**6a**): To a suspension of **5a** (930 mg, 2.0 mmol), diethyl(3-pyridyl)borane (441 mg, 3.0 mmol) and bis(triphenylphosphine)palladium(II) dichloride (28 mg, 0.040 mmol) in THF (10 mL) were added aqueous Na_2CO_3 (2 M, 4 mL, 8 mmol) at room temperature. The reaction mixture was stirred at 80 °C for 3 h, cooled, and added with H_2O. The resulting mixture was extracted twice with EtOAc, dried over Na_2SO_4, and concentrated in vacuo. The obtained crude residue was used for the next step without further purification. To a solution of the mixture in MeOH (50 mL) was added a solution of KOH (2.81 g, 50 mmol) in MeOH (30 mL) at room temperature. The reaction mixture was stirred for 2 h at room temperature, and the solvent was removed under reduced pressure. After addition of H_2O, the resulting mixture was extracted twice with CH_2Cl_2, dried over Na_2SO_4, and concentrated in vacuo. The residue was purified by column chromatography on silica gel (*n*-hexane:ethyl acetate = 1:1) to afford **6a** (670 mg, 95%) as a white solid. ^{1}H NMR (400 MHz, CDCl3) δ 8.61 (d, *J* = 1.9 Hz, 1H), 8.45 (dd, *J* = 4.8, 1.9 Hz, 1H), 7.64 (ddd, *J* = 7.9, 1.9, 1.9 Hz, 1H), 7.21 (dd, *J* = 7.9, 4.8 Hz, 1H), 5.97 (dd, *J* = 3.2, 1.7 Hz, 1H), 3.66–3.56 (m, 1H), 2.24 (ddd, *J* = 15.8, 6.3, 3.2 Hz, 1H), 2.08–1.96 (m, 2H), 1.86–1.23 (m, 14H), 1.21–1.10 (m, 1H), 1.07–0.95 (m, 5H), 0.86 (s, 3H), 0.81–0.72 (m, 1H); ^{13}C NMR (100 MHz, $CDCl_3$) δ 151.7, 147.9, 147.8, 133.6, 133.0, 129.2, 123.0, 71.2, 57.4, 54.6, 47.5, 45.0, 38.2, 36.8, 35.7, 35.3, 34.0, 31.9, 31.7, 31.5, 28.6, 21.2, 16.7, 12.3; IR (ATR): 3332, 2927, 796, 710 cm^{-1}; mp 214–216 °C (EtOAc); $[\alpha]_D^{20}$ +36.9 (c 1.00, $CHCl_3$); HRMS (ESI) *m/z* $[M + H]^+$ calcd for $C_{24}H_{33}NO$ 352.2635; found 352.2640.

17-(3-Pyridyl)-5α-androsta-16-ene-3-one (**7a**): To a solution of **6a** (106 mg, 0.3 mmol) in 10 mL of CH_2Cl_2 was added Dess–Martin periodinane (191 mg, 0.45 mmol) at 0 °C. The reaction mixture was stirred at room temperature for 1 h and added with H_2O. Subsequently, the mixture was extracted twice with EtOAc, dried over Na_2SO_4, and concentrated in vacuo. The residue was purified by column chromatography on silica gel (*n*-hexane:ethyl acetate = 2:1–1:1) to afford **7a** (103 mg, 98%) as a white solid. ^{1}H NMR (400 MHz, $CDCl_3$) δ 8.62 (d, *J* = 1.9 Hz, 1H), 8.46 (dd, *J* = 4.8, 1.9 Hz, 1H), 7.65 (ddd, *J* = 7.9, 1.9, 1.9 Hz, 1H), 7.23 (dd, *J* = 7.9, 4.8 Hz, 1H), 5.99 (dd, *J* = 3.2, 1.7 Hz, 1H), 2.46–2.21 (m, 4H), 2.15–1.98 (m, 4H), 1.84–1.31 (m, 10H), 1.12–0.99 (m, 7H), 0.92-0.83 (m, 1H); ^{13}C NMR (100 MHz, $CDCl_3$) δ 211.9, 151.6, 147.8, 133.7, 132.9, 129.2, 123.0, 57.2, 54.0, 47.5, 46.8, 44.7, 38.3, 38.1, 35.8, 35.2, 33.9, 31.7, 31.5, 28.8, 21.3, 16.7, 11.4; IR (ATR): 2941, 1707, 793, 709 cm^{-1}; mp 204–206 °C (EtOAc); $[\alpha]_D^{20}$ +69.9 (c 1.00, $CHCl_3$); HRMS (ESI) *m/z* $[M + H]^+$ calcd for $C_{24}H_{31}NO$ 350.2478; found 350.2482.

3α-Acetoxy-5β-androsta-16-ene-17-yl-trifluoromethanesulfonate (**5b**): The experimental procedure for the synthesis of **5b** from 3β-hydroxy-5α-androstan-17-one (**4b**, 871 mg, 3.0 mmol) was identical to that for **5a**. Purification by column chromatography on silica gel (*n*-hexane:ethyl acetate = 20:1) afforded **5b** (983 mg, 71%) as a colorless oil. ^{1}H NMR (400 MHz, $CDCl_3$) δ 5.59–5.54 (m, 1H), 4.78–4.66 (m, 1H), 2.21 (ddd, *J* = 14.8, 5.4, 3.4 Hz, 1H), 2.09–0.84 (m, 28H); ^{13}C NMR (100 MHz, $CDCl_3$) δ 170.6, 159.2, 118.5 (q, $^1J_{C\text{-}F}$ = 320 Hz), 114.5, 74.0, 54.3, 44.8, 41.8, 41.0, 34.9, 34.8, 33.8, 32.8, 32.2, 28.5, 26.6, 26.5, 25.2, 23.1, 21.4, 20.0, 15.2; ^{19}F NMR (376 MHz, $CDCl_3$) δ –73.5; IR (ATR): 2941, 1734, 1421, 1207, 755 cm^{-1}; $[\alpha]_D^{20}$ +44.2 (c 1.00, $CHCl_3$); HRMS (EI) *m/z* $[M]^+$ calcd for $C_{22}H_{31}F_3O_5S$ 464.1844; found 464.1842.

17-(3-Pyridyl)-5β-androsta-16-ene-3α-ol (**6b**): The experimental procedure for the synthesis of **6b** from **5b** (930 mg, 2.0 mmol) was identical to that for **6a**. Purification by column chromatography on silica gel (*n*-hexane:ethyl acetate = 1:1) afforded **6b** (548 mg, 78%) as a white solid. ^{1}H NMR (400 MHz, $CDCl_3$) δ 8.63–8.59 (m, 1H), 8.48–8.43 (m, 1H), 7.64 (ddd, *J* = 7.9, 1.9, 1.9 Hz, 1H), 7.22 (dd, *J* = 7.9, 4.8 Hz, 1H), 5.97 (dd, *J* = 3.2, 1.7 Hz, 1H), 3.70–3.60 (m, 1H), 2.24 (ddd, *J* = 15.8, 6.2, 3.2 Hz, 1H), 2.07–1.86 (m, 3H), 1.85–1.17 (m, 16H), 1.04–0.95 (m, 7H); ^{13}C NMR (100 MHz, $CDCl_3$) δ 151.7, 147.9, 147.8, 133.7, 133.0, 129.2, 123.0, 71.7, 57.5, 47.6, 42.1, 40.8, 36.5, 35.5, 35.2, 34.8, 34.4, 31.7, 30.5, 27.1, 26.4, 23.3, 20.7, 16.6; IR (ATR): 3322, 2927, 798, 710 cm^{-1}; mp 179–182 °C (EtOAc); $[\alpha]_D^{20}$ +61.8 (c 1.00, $CHCl_3$); HRMS (ESI) *m/z* $[M + H]^+$ calcd for $C_{24}H_{33}NO$ 352.2634; found 352.2642.

17-(3-Pyridyl)-5β-androsta-16-ene-3-one (**7b**): The experimental procedure for the synthesis of **7b** from **6b** (106 mg, 0.3 mmol) was identical to that for **7a**. Purification by column chromatography on silica gel (*n*-hexane:ethyl acetate = 2:1–1:1) afforded **7b** (98 mg, 94%) as a white solid. ^{1}H NMR (400 MHz, $CDCl_3$) δ 8.64-8.60 (m, 1H), 8.49-8.45 (m, 1H), 7.67-7.62 (m, 1H), 7.23 (dd, *J* = 7.8, 4.8 Hz, 1H), 6.01-5.98 (m, 1H), 2.73 (dd, *J* = 14,3, 14.3 Hz, 1H), 2.40-1.18 (m, 19H), 1.08 (s, 3H), 1.04 (s, 3H); ^{13}C NMR (100 MHz, $CDCl_3$) δ 213.2, 151.6, 147.9, 147.8, 133.7, 132.8, 129.2, 123.0, 57.4, 47.6, 44.3, 42.3, 41.1, 37.1, 36.9, 35.4, 35.1, 34.1, 31.6, 26.5, 25.7, 22.6, 21.1, 16.7; IR (ATR): 2929, 1712, 801, 714 cm^{-1}; mp 162–164 °C (EtOAc); $[\alpha]_D^{20}$ +76.3 (c 1.00, $CHCl_3$); HRMS (ESI) *m/z* $[M + H]^+$ calcd for $C_{24}H_{31}NO$ 350.2478; found 350.2475.

3. Copies of NMR charts

^{1}H NMR (400 MHz, $CDCl_3$)

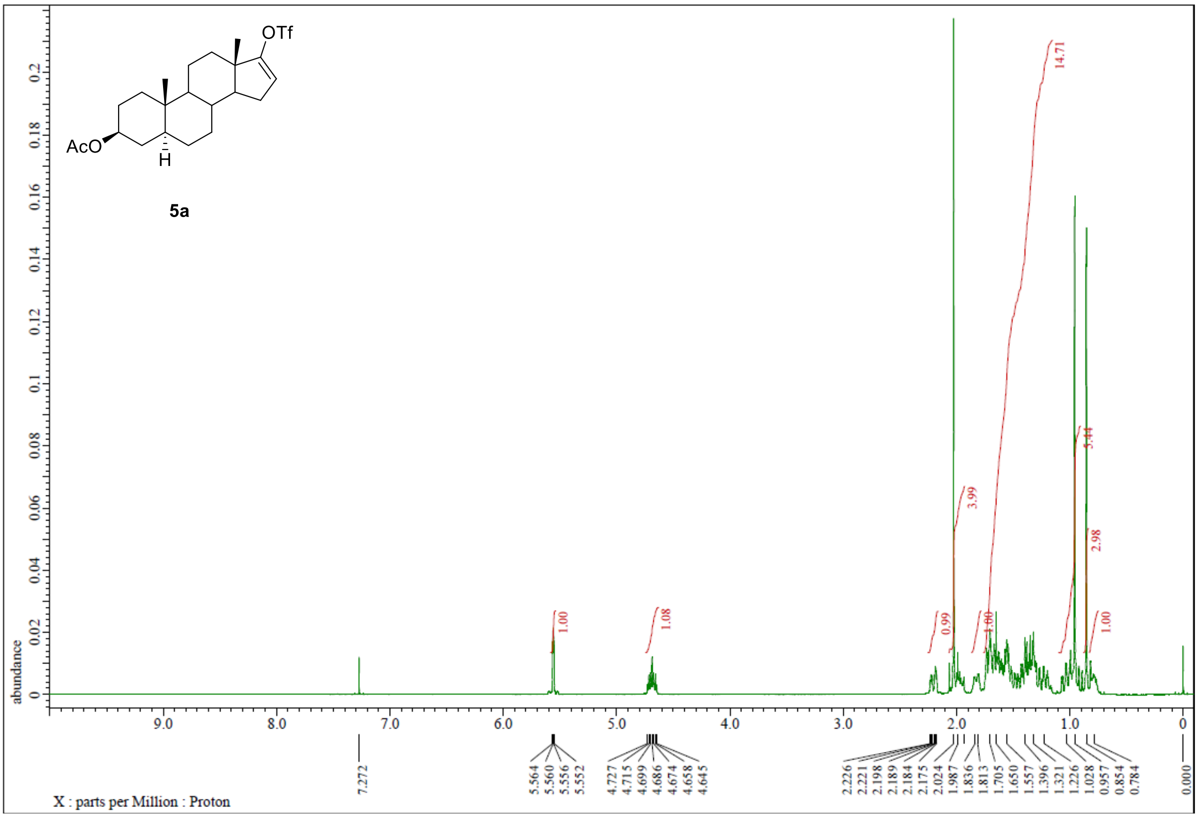

^{13}C NMR (100 MHz, $CDCl_3$)

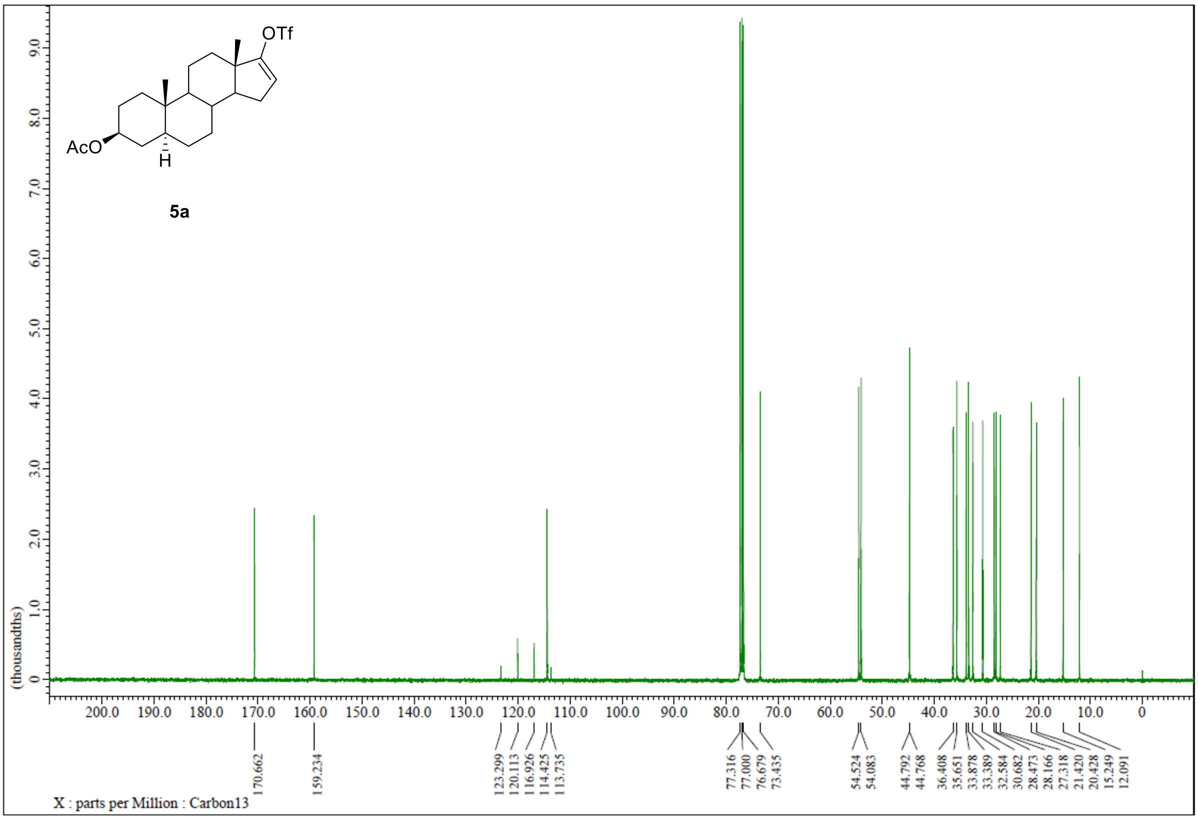

^{19}F NMR (376 MHz, $CDCl_3$)

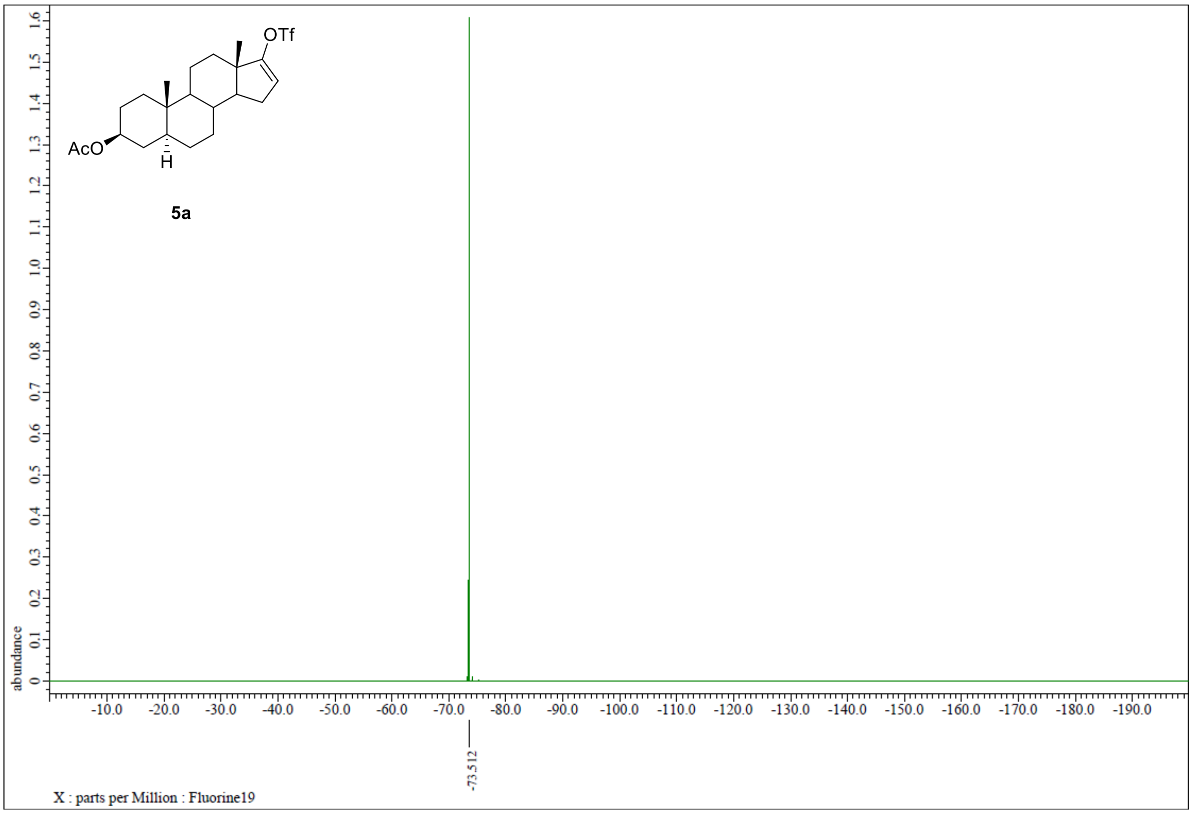

^1H NMR (400 MHz, $CDCl_3$)

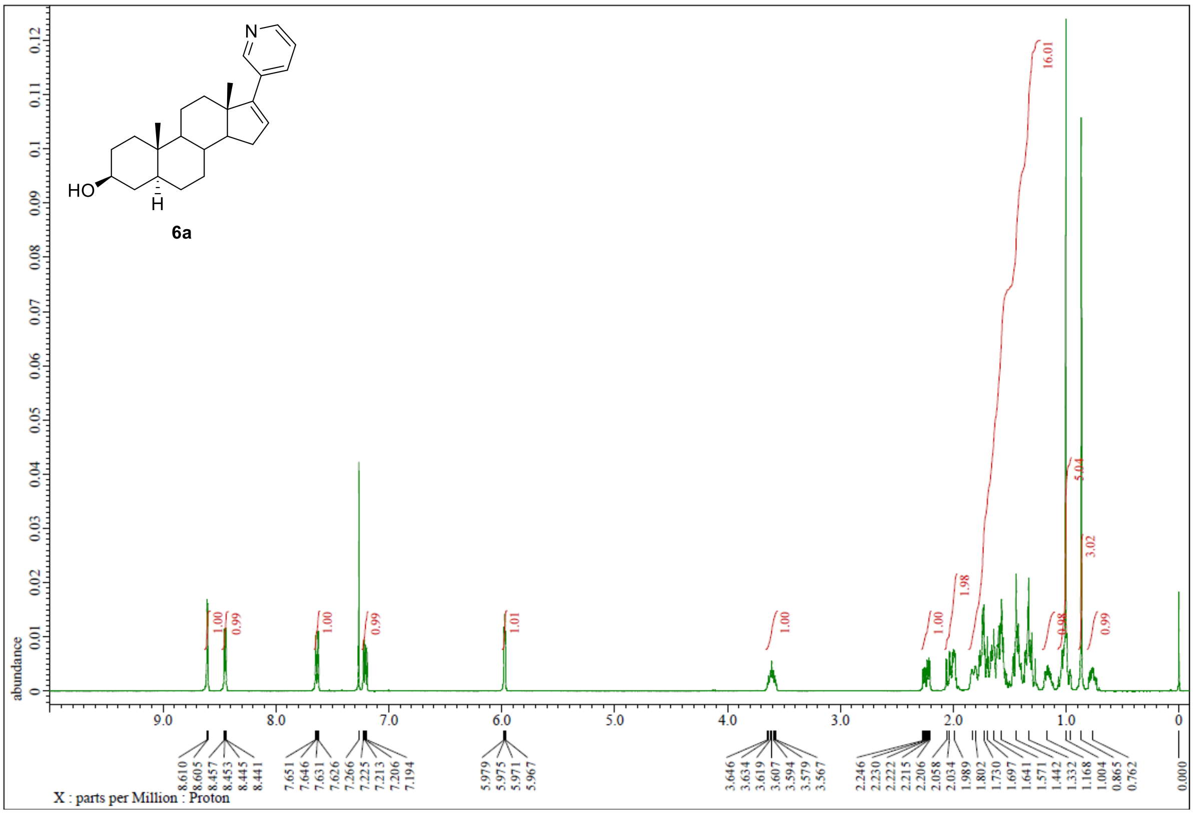

^{13}C NMR (100 MHz, $CDCl_3$)

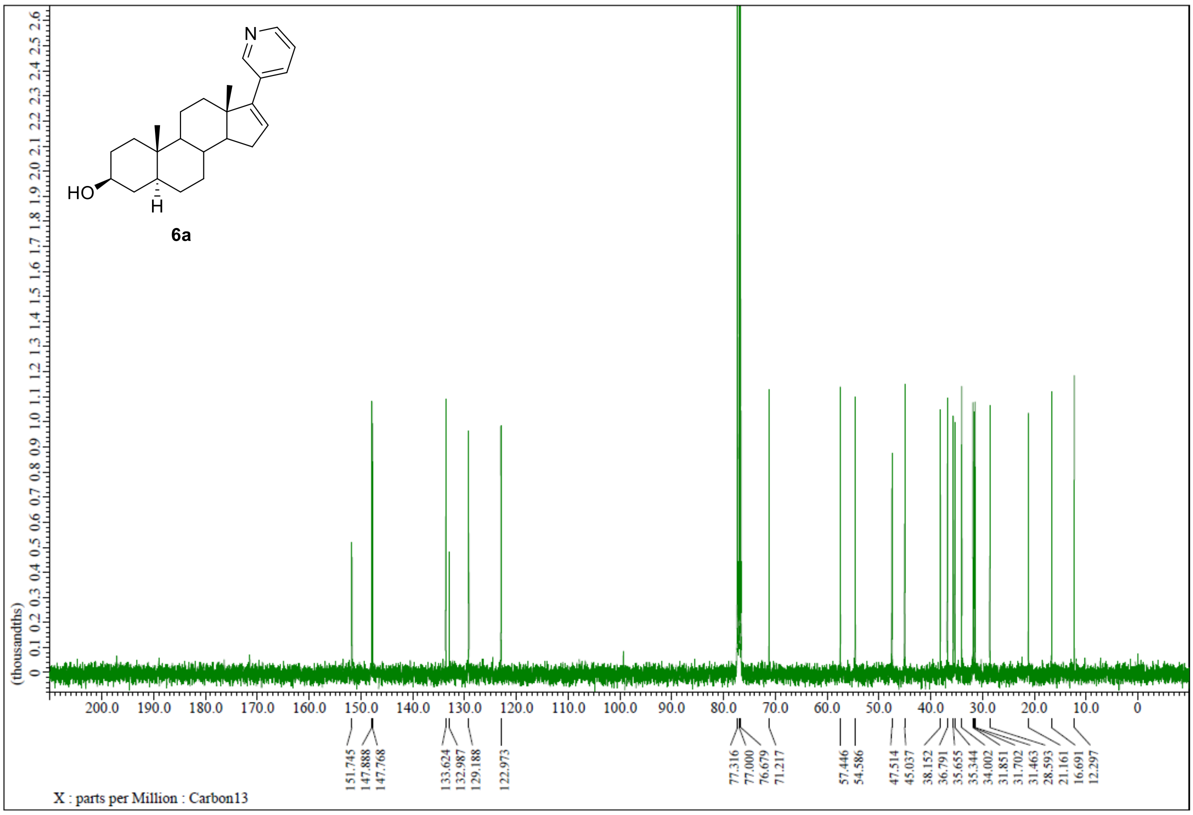

^{1}H NMR (400 MHz, $CDCl_3$)

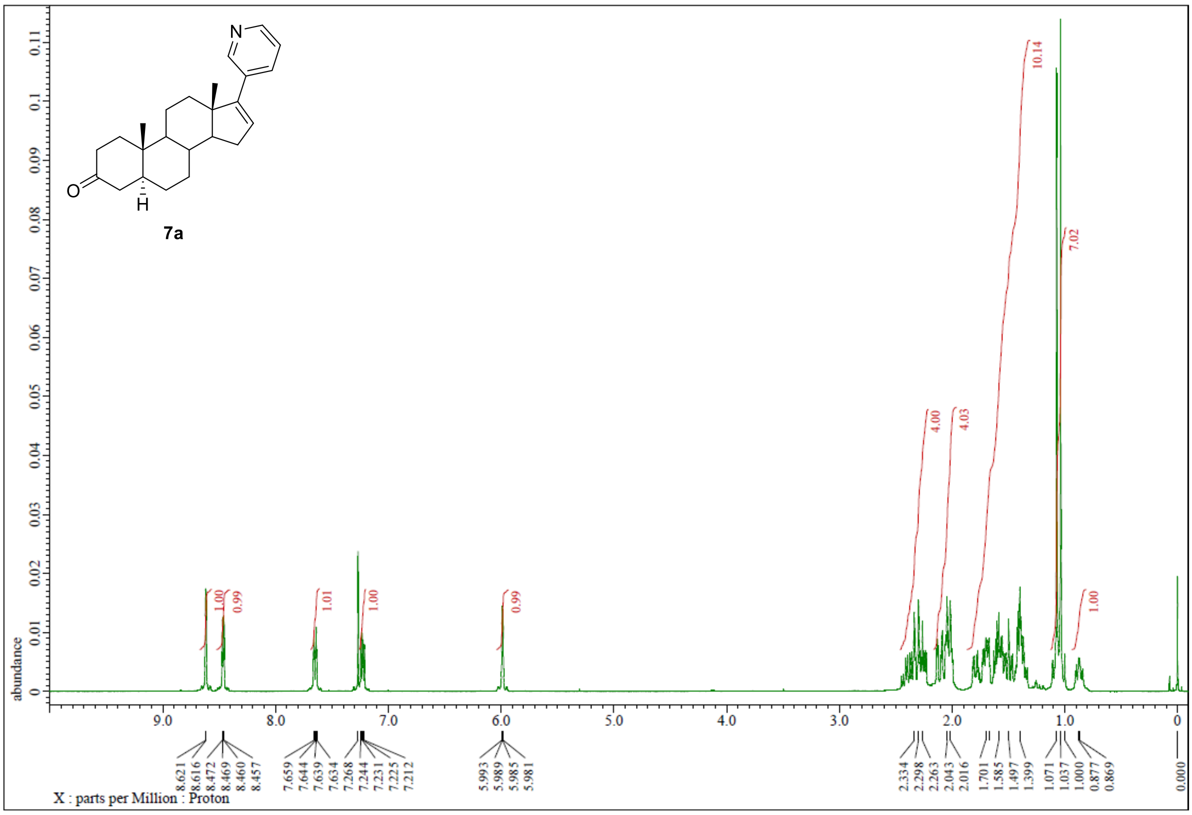

^{13}C NMR (100 MHz, $CDCl_3$)

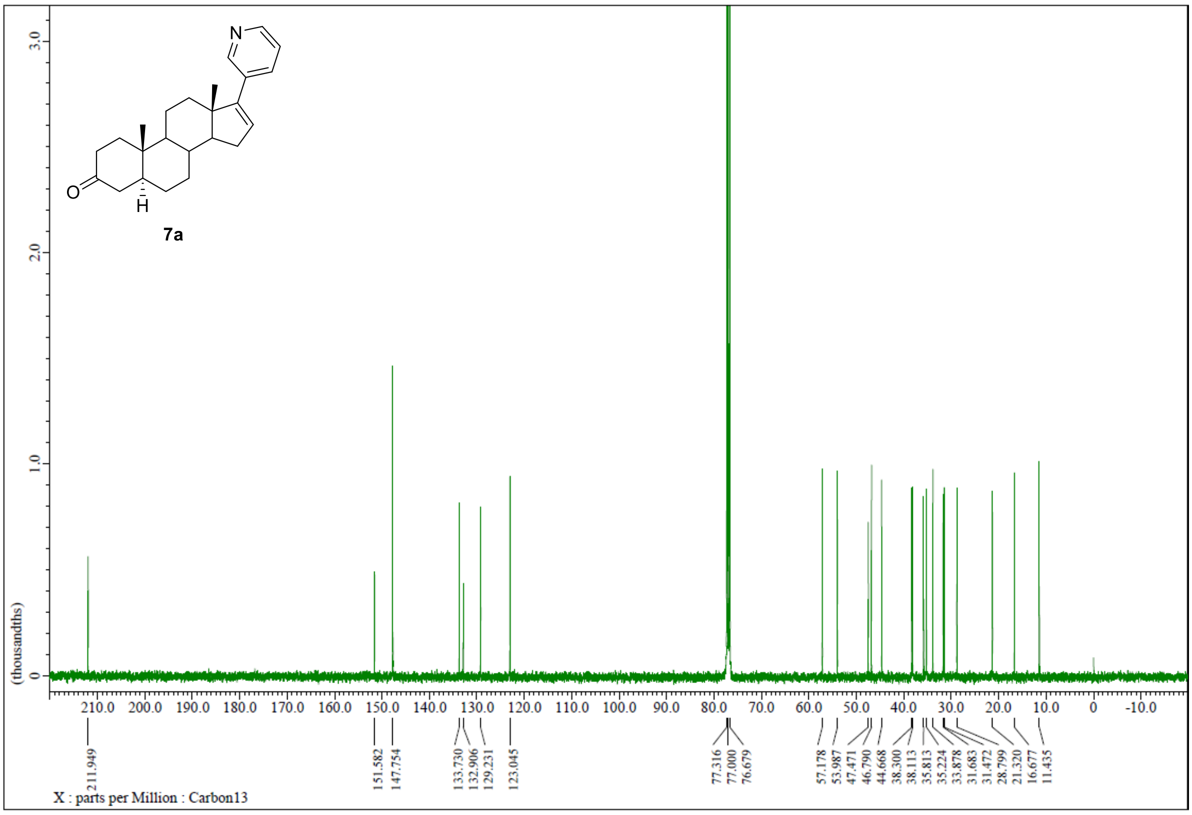

^1H NMR (400 MHz, $CDCl_3$)

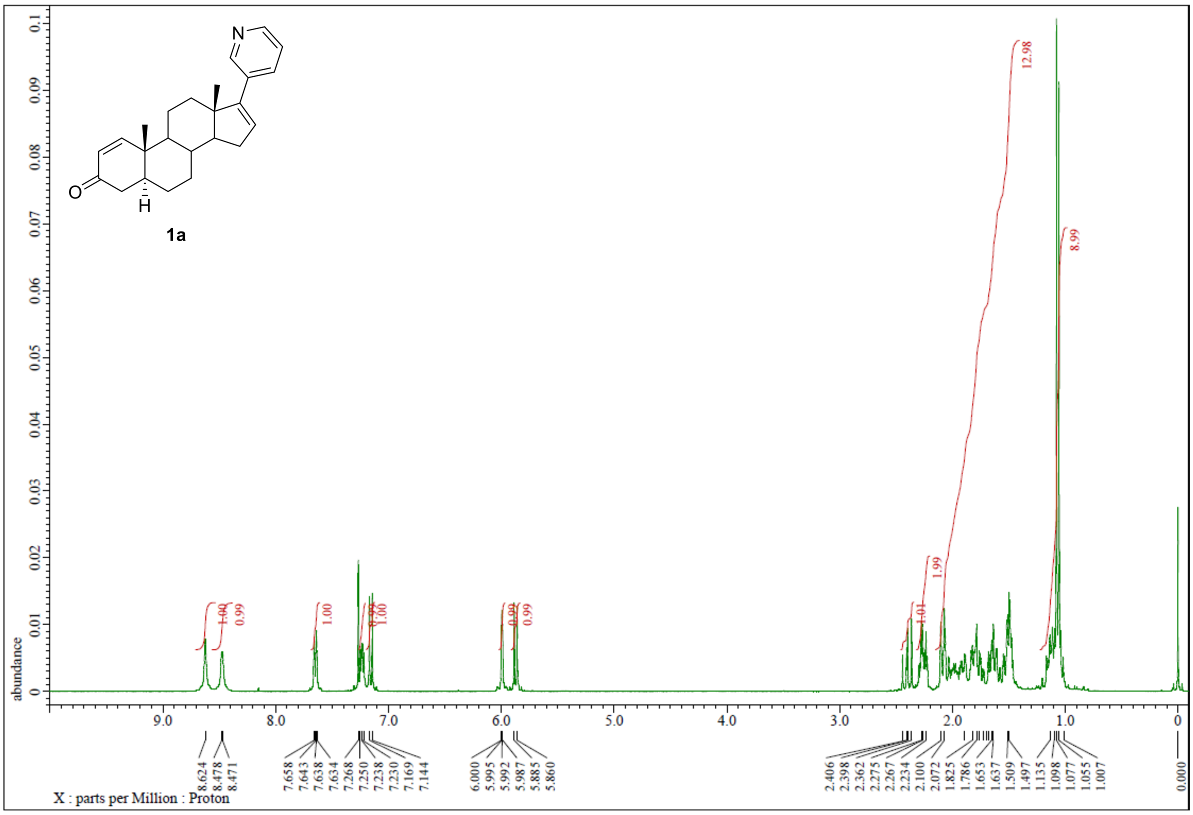

^{13}C NMR (100 MHz, $CDCl_3$)

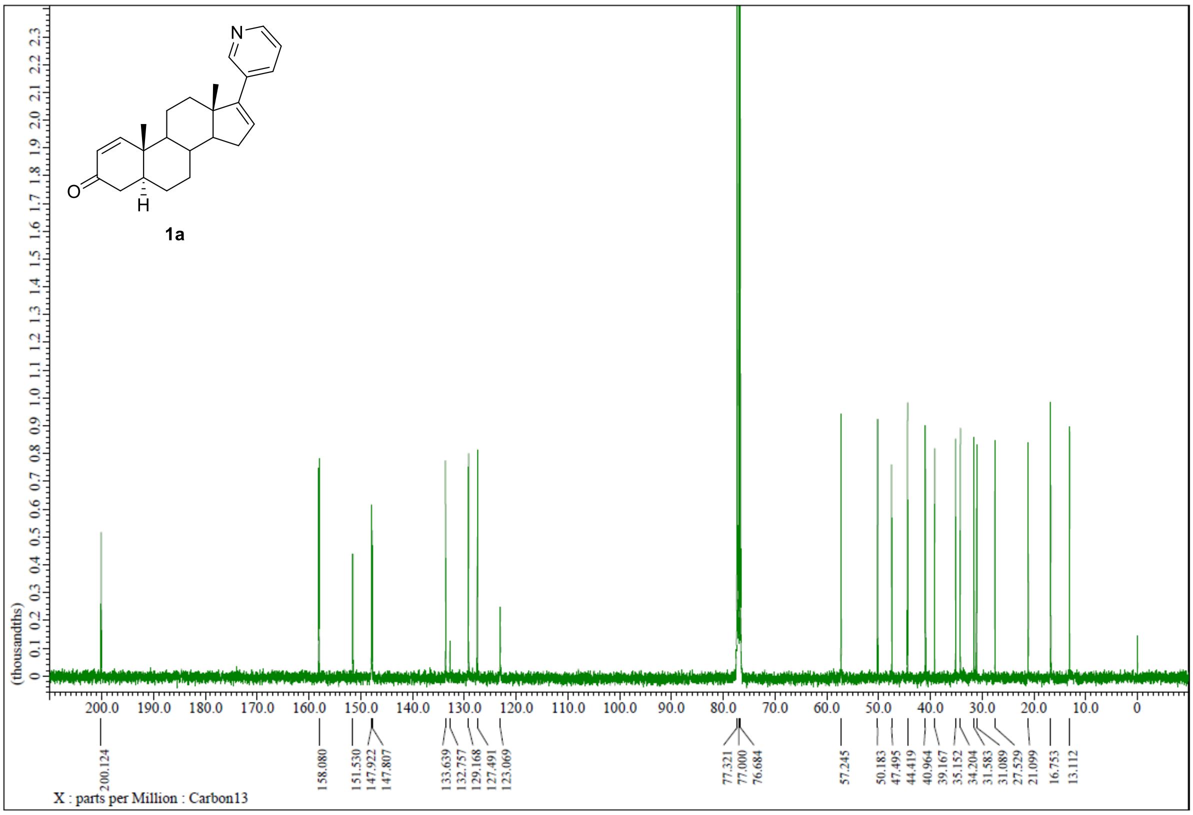

COSY

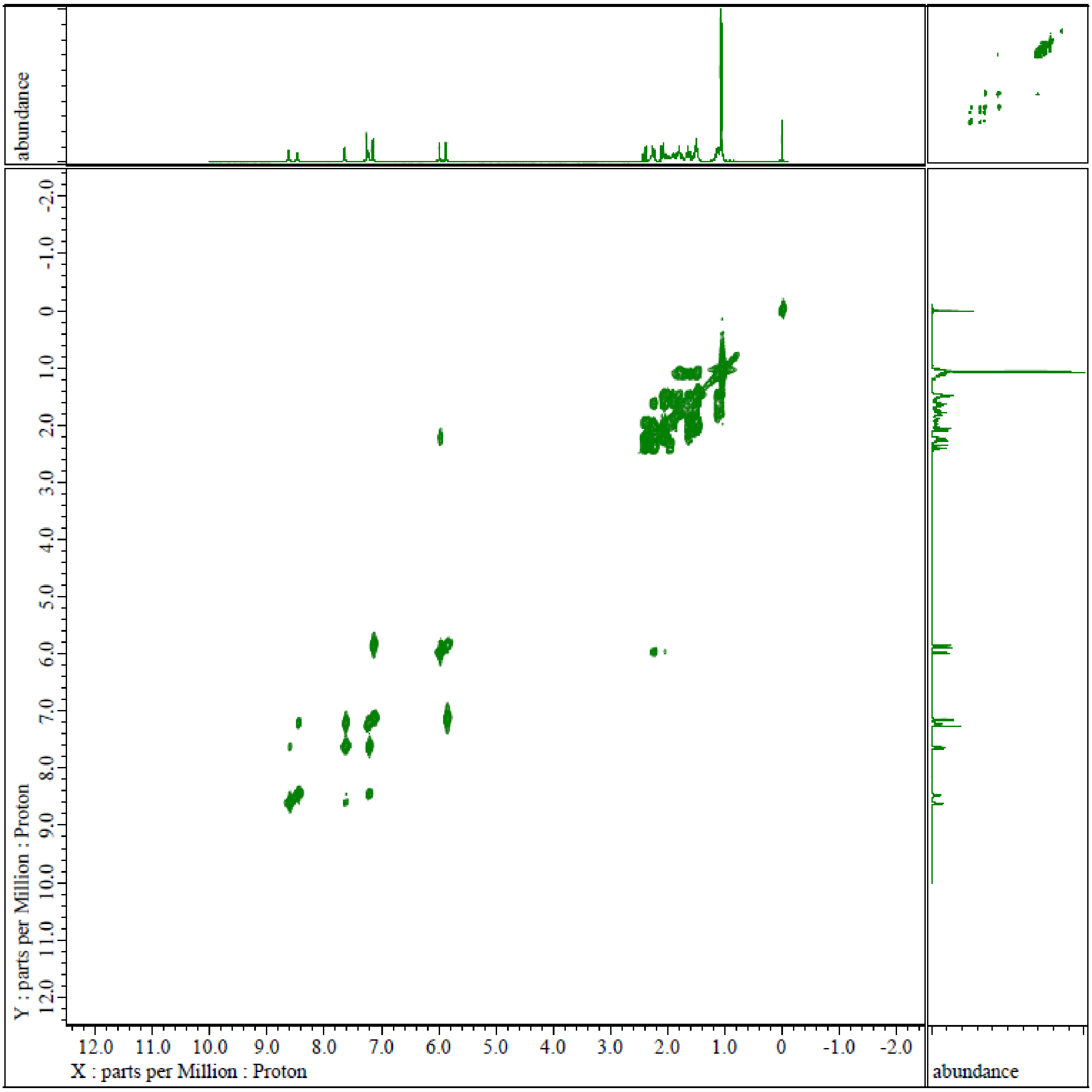
abundance
Y : parts per Million : Proton
X : parts per Million : Proton
12.0
11.0
10.0
9.0
8.0
7.0
6.0
5.0
4.0
3.0
2.0
1.0
0
-1.0
-2.0
abundance

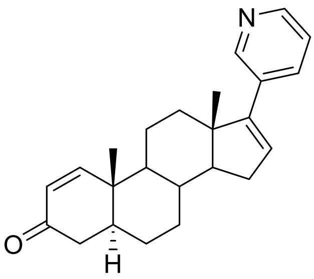
N
O
H

1a

NOESY

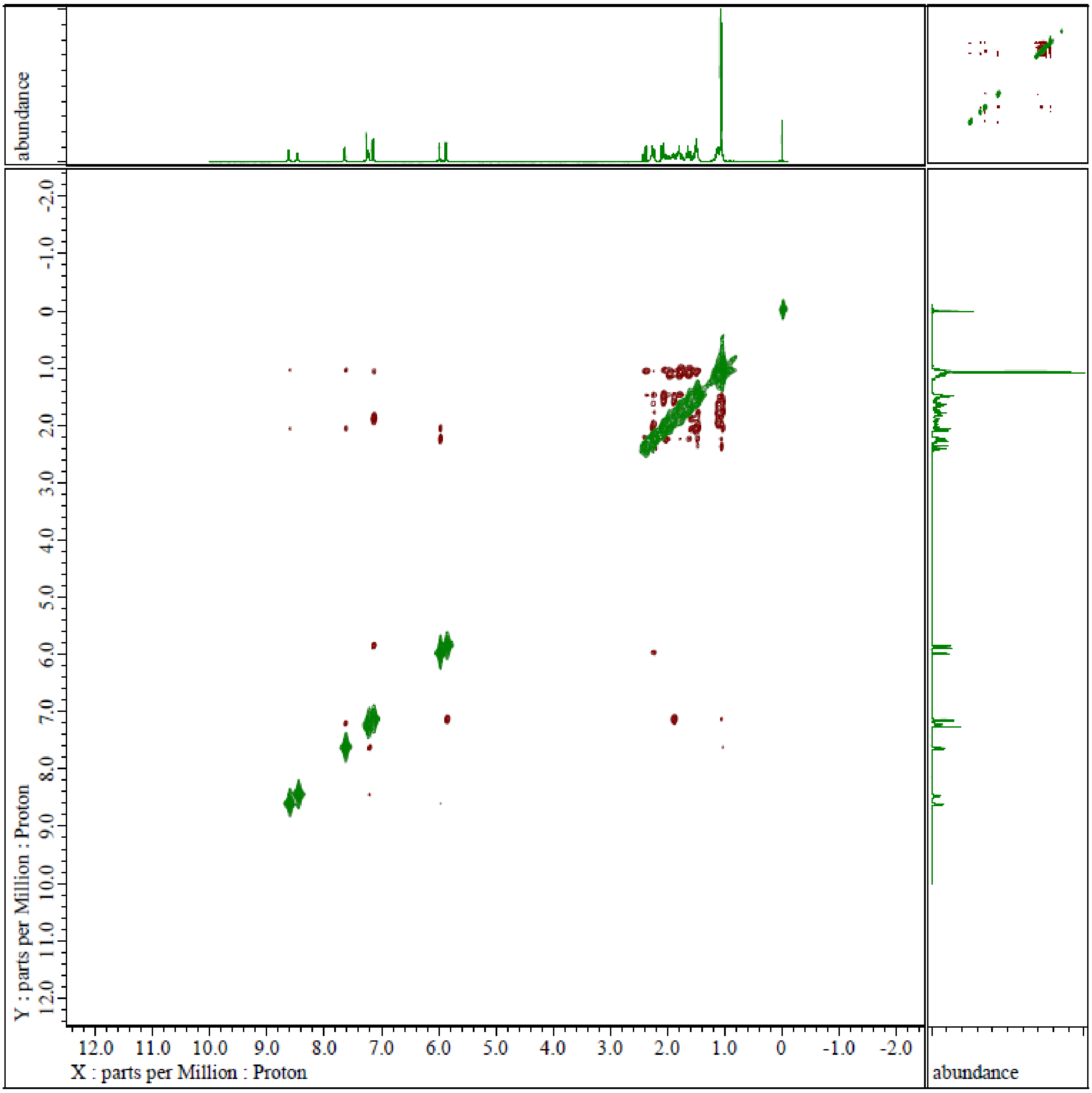

abundance
Y : parts per Million : Proton
X : parts per Million : Proton
12.0
11.0
10.0
9.0
8.0
7.0
6.0
5.0
4.0
3.0
2.0
1.0
0
-1.0
-2.0
abundance

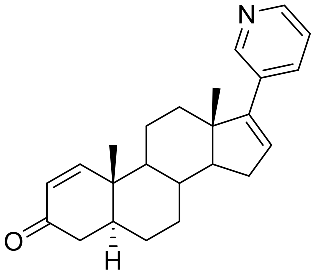

N
O
H

1a

^{1}H NMR (400 MHz, $CDCl_3$)

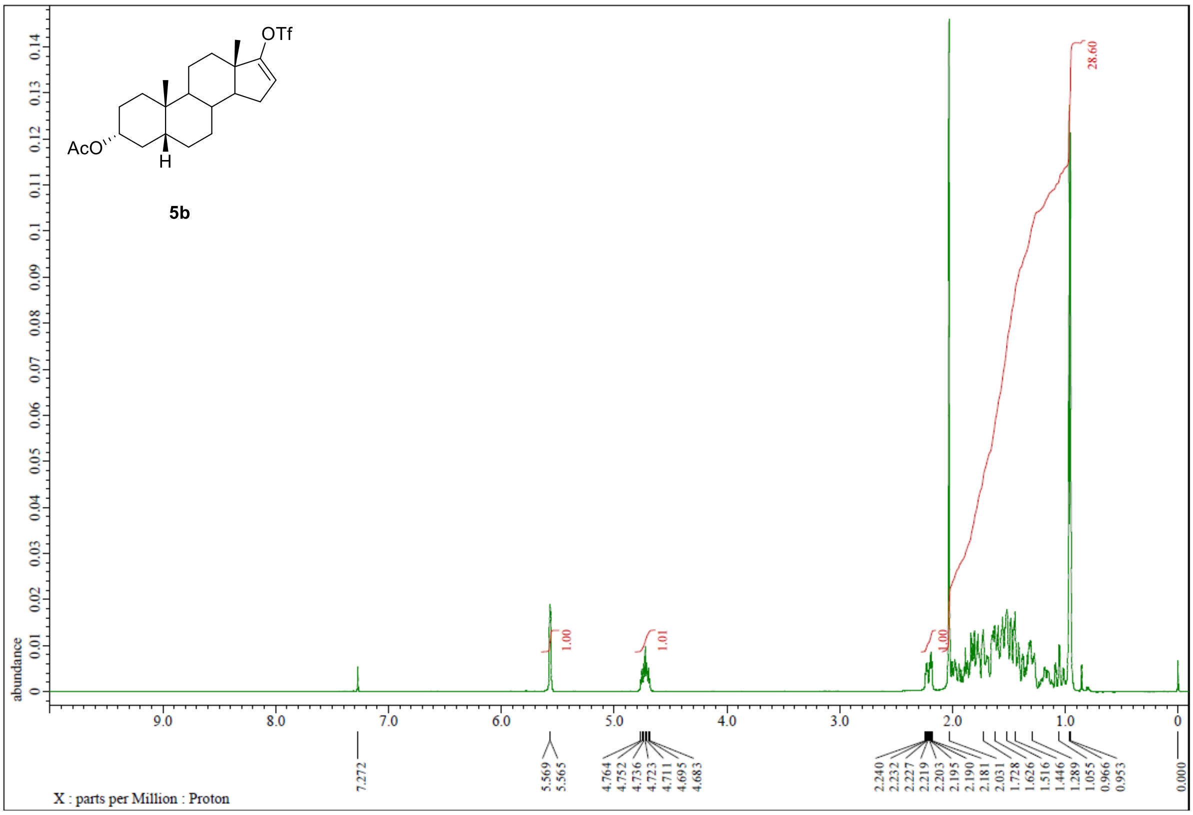

^{13}C NMR (100 MHz, $CDCl_3$)

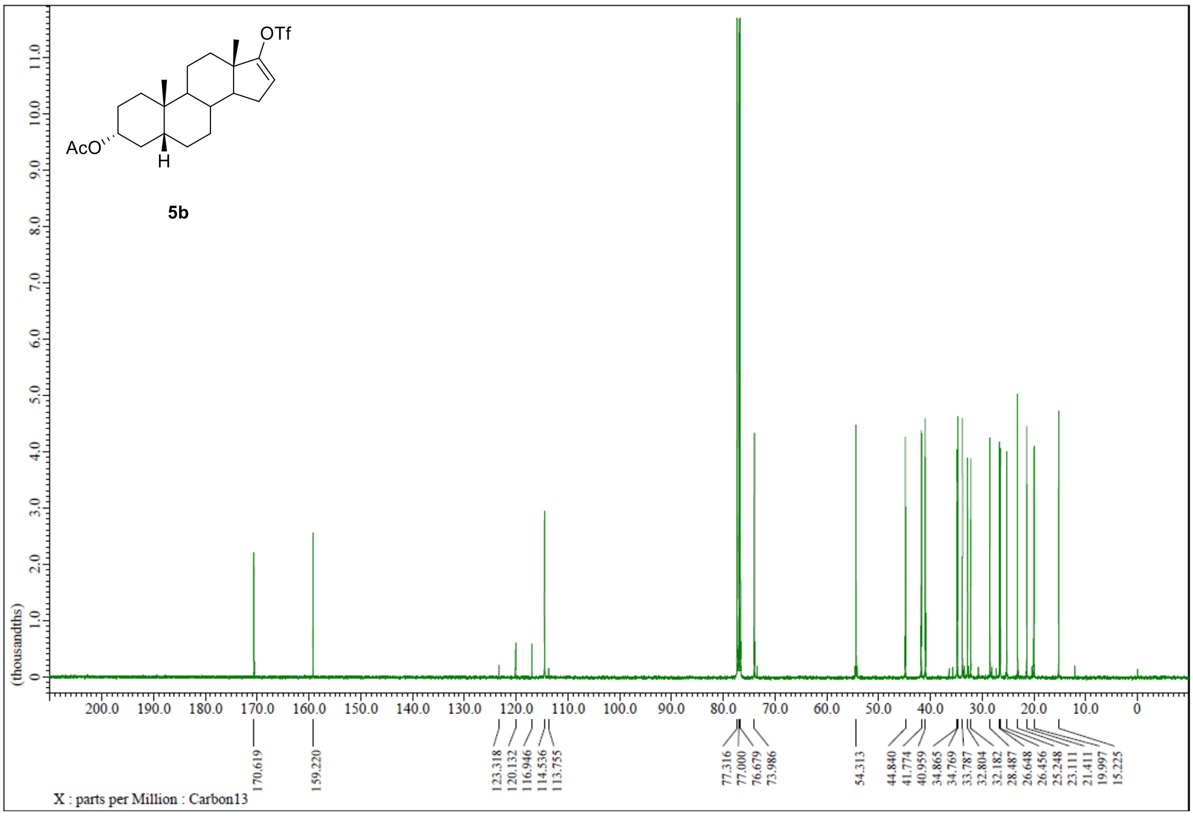

^{19}F NMR (376 MHz, $CDCl_3$)

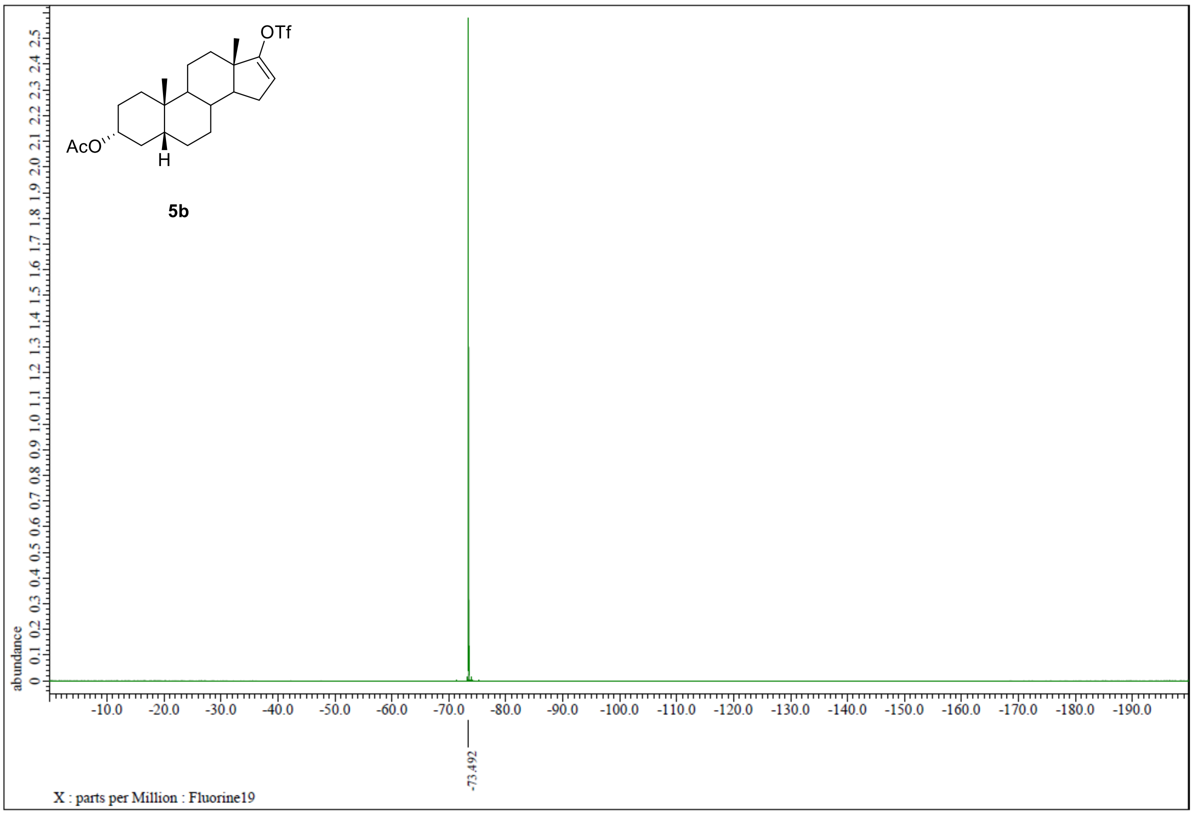

^{1}H NMR (400 MHz, $CDCl_3$)

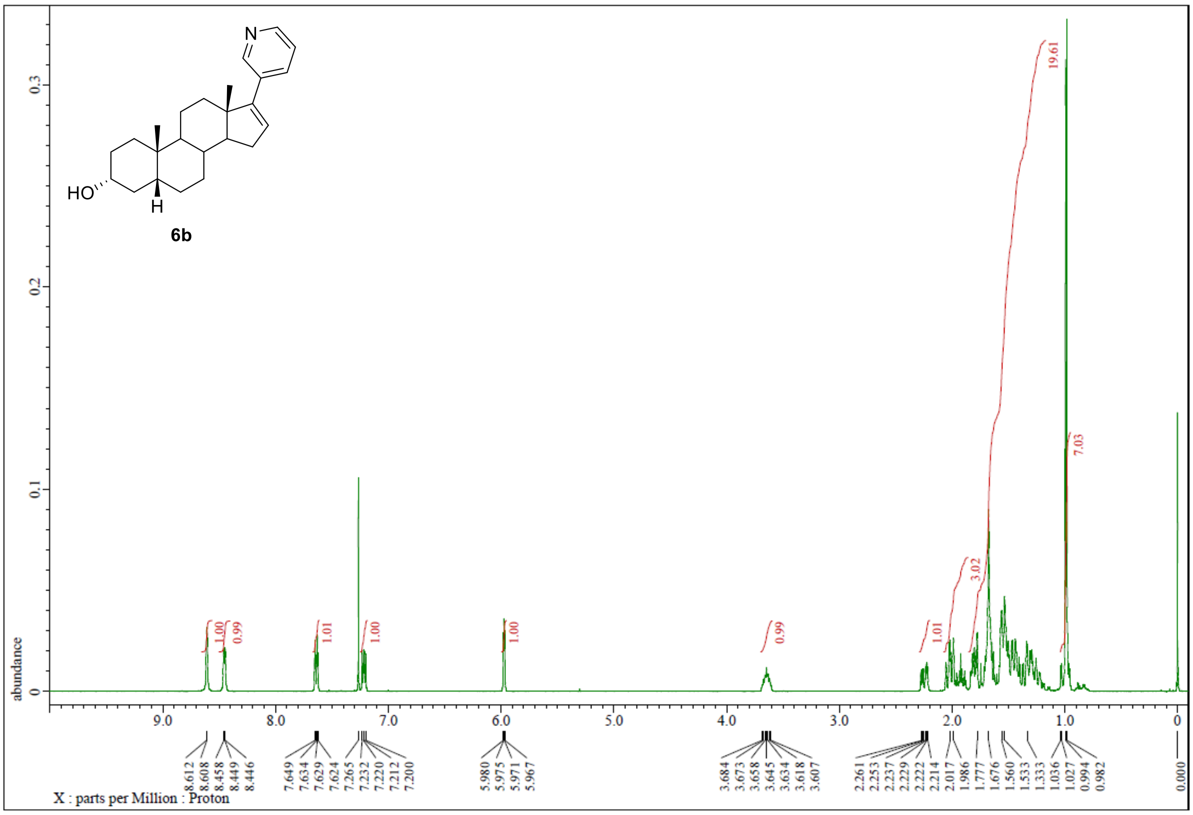

^{13}C NMR (100 MHz, $CDCl_3$)

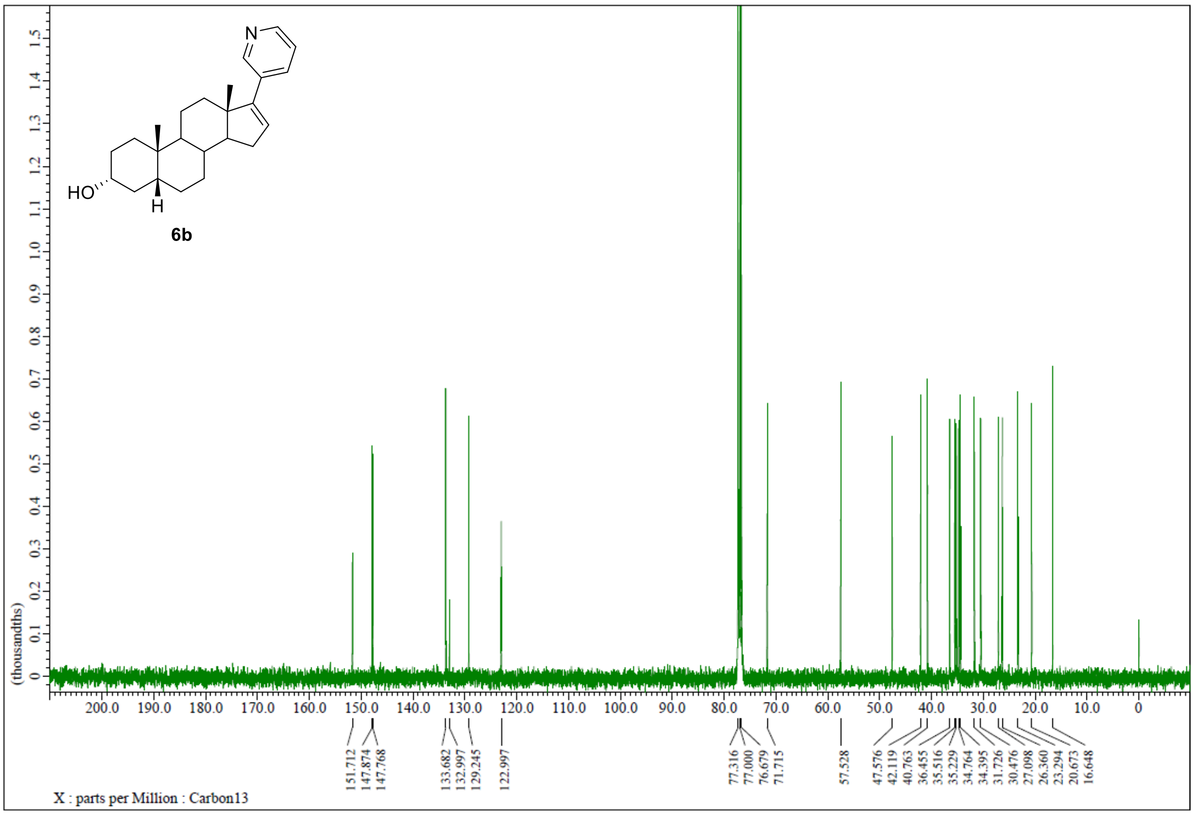

^1H NMR (400 MHz, $CDCl_3$)

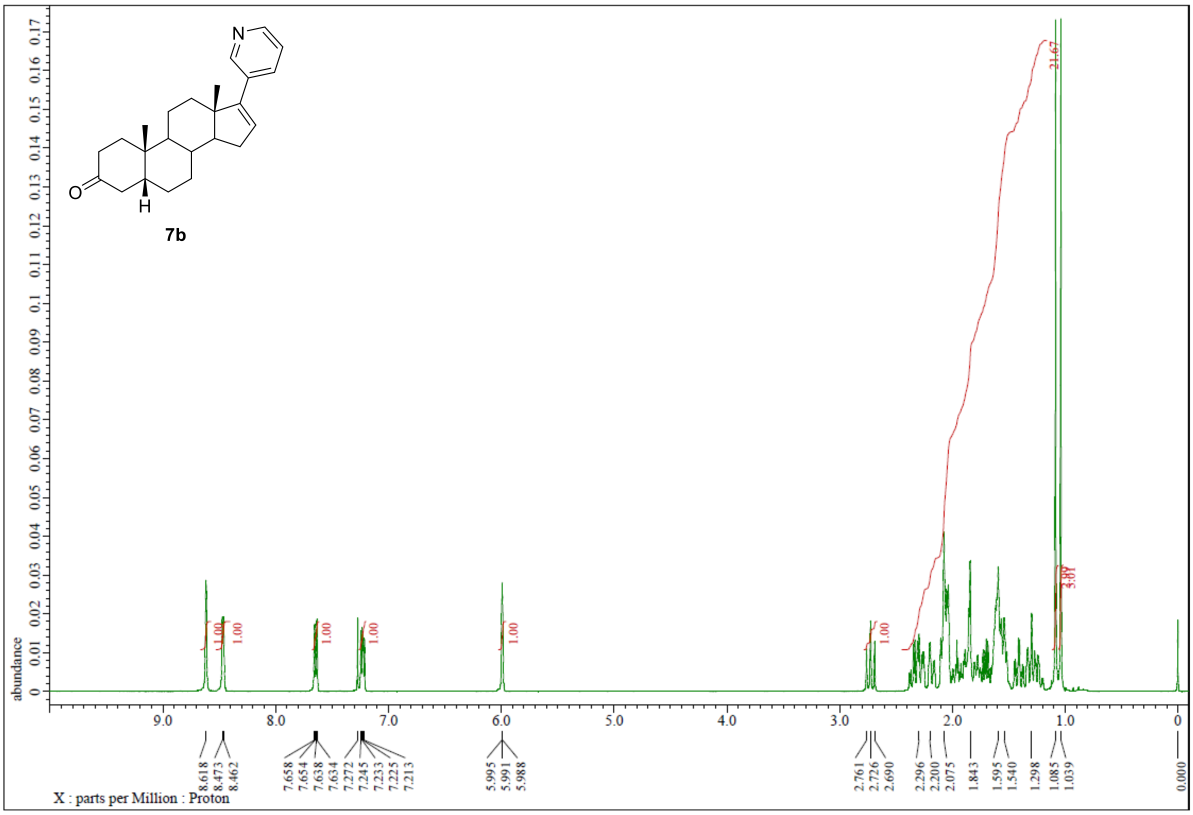

^{13}C NMR (100 MHz, $CDCl_3$)

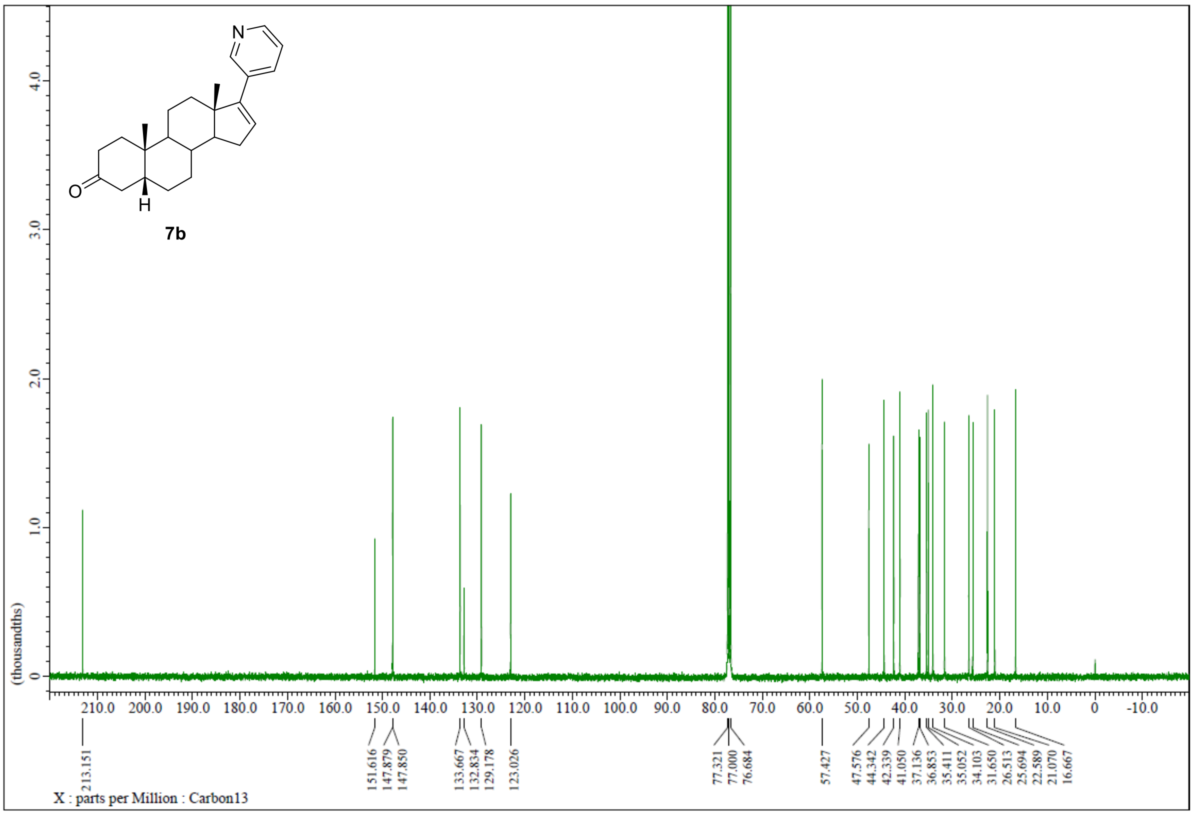

^{1}H NMR (400 MHz, $CDCl_3$)

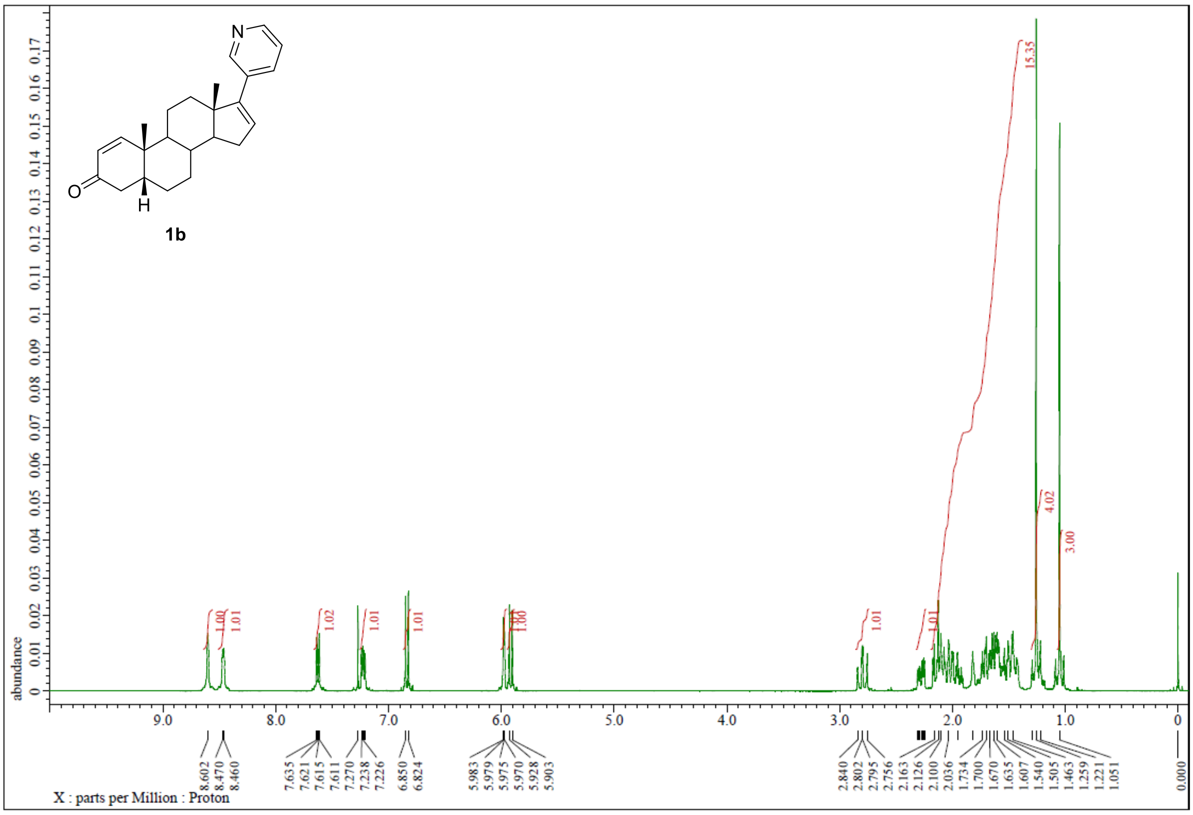

^{13}C NMR (100 MHz, $CDCl_3$)

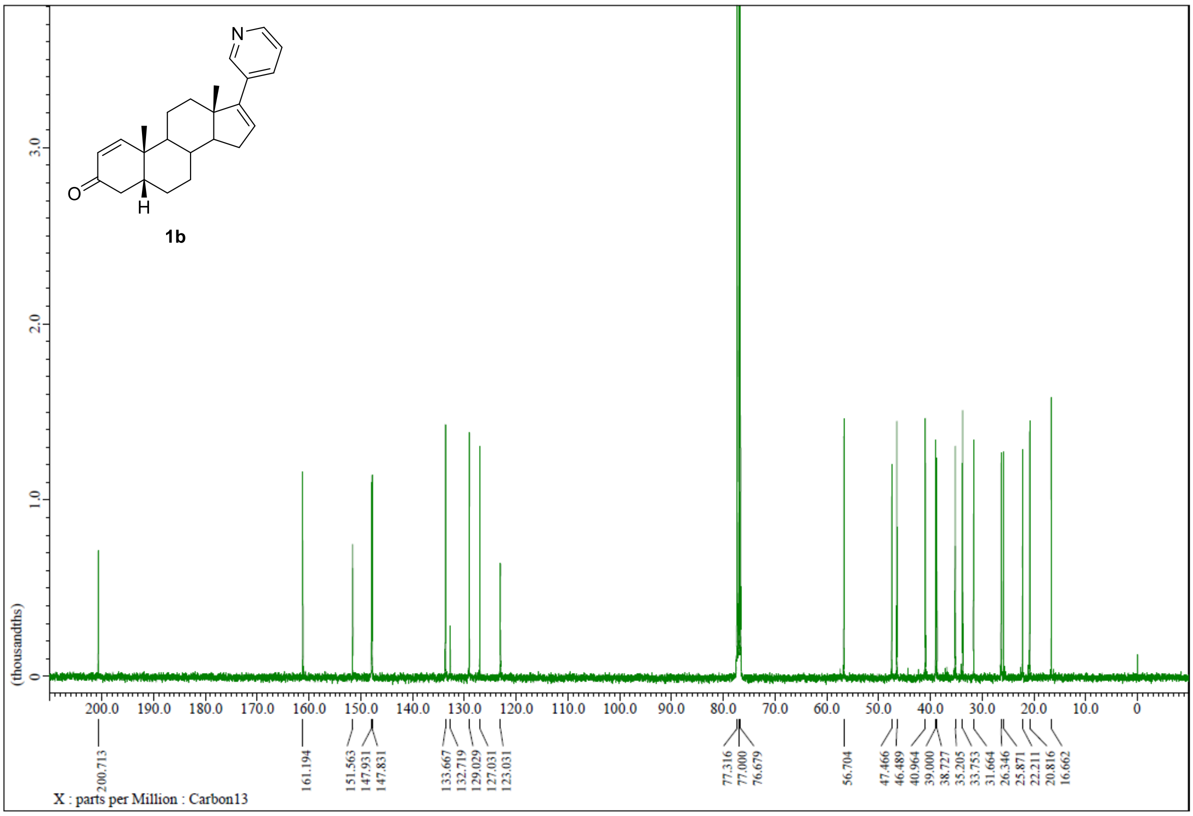

COSY

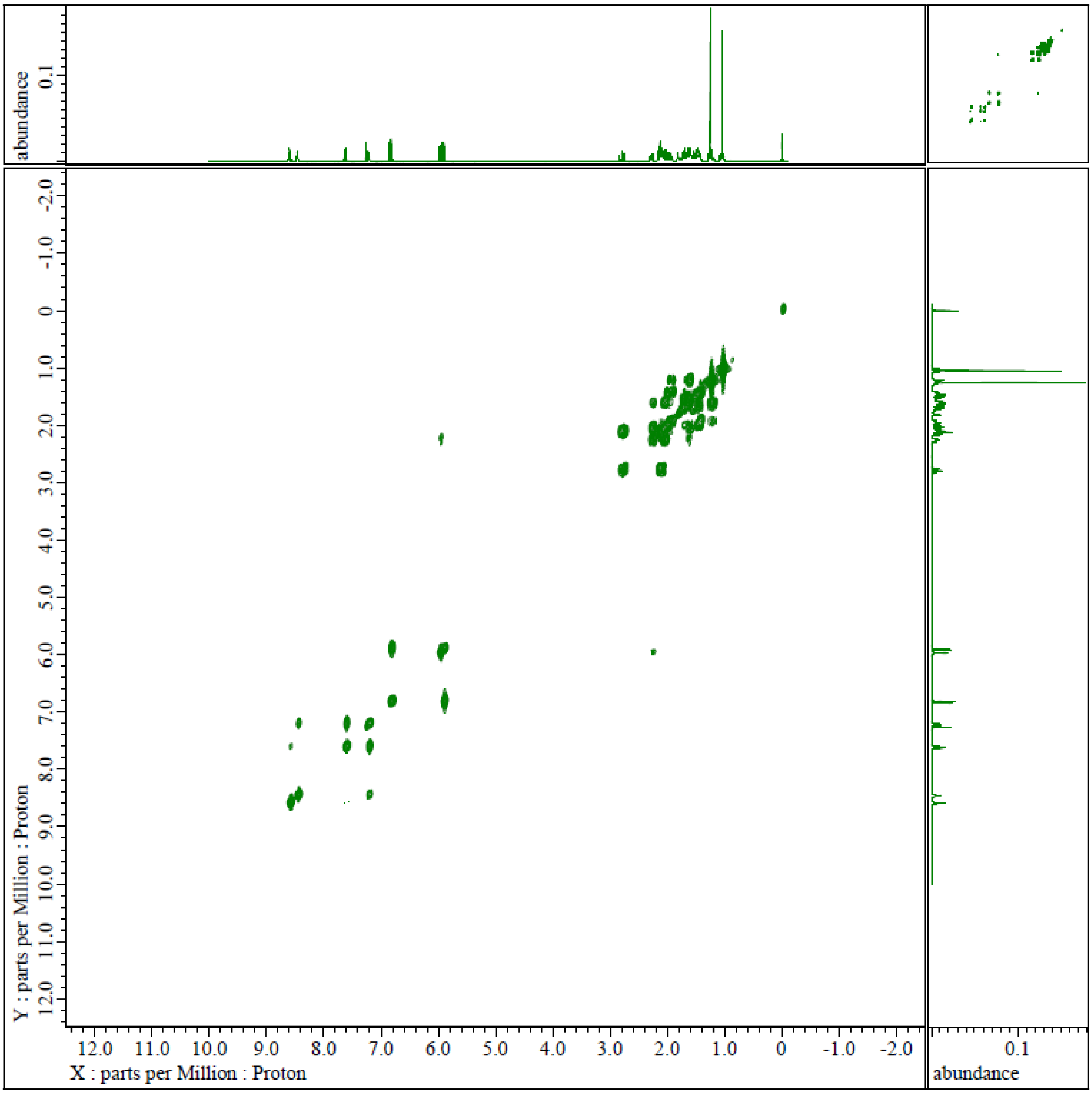
abundance
0.1
Y : parts per Million : Proton
X : parts per Million : Proton
12.0
11.0
10.0
9.0
8.0
7.0
6.0
5.0
4.0
3.0
2.0
1.0
0
-1.0
-2.0
abundance

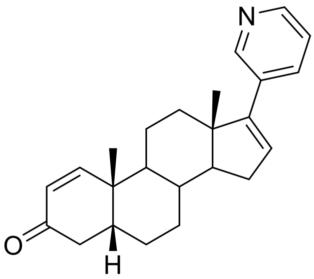
N
O
H

1b

NOESY

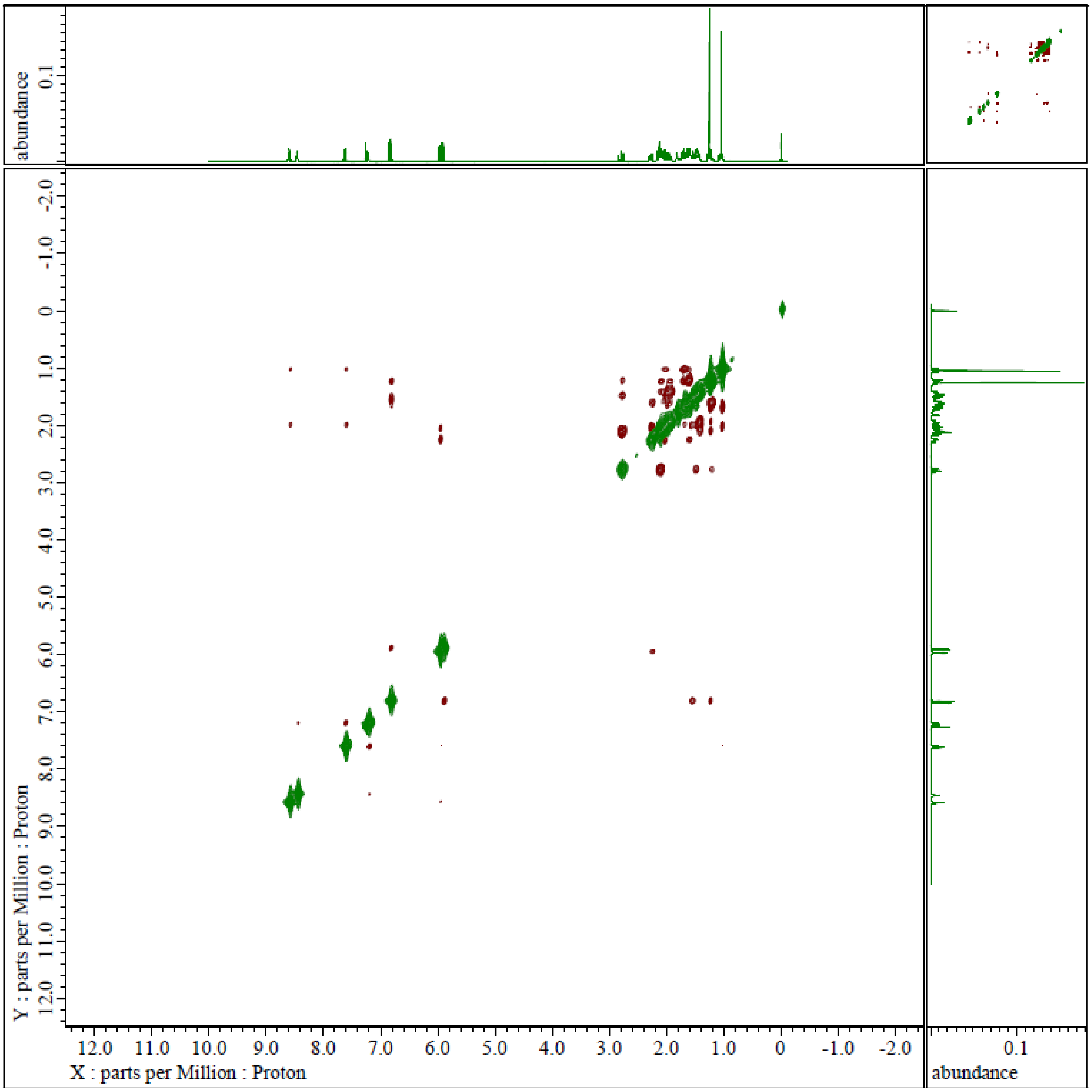

abundance
0.1
-2.0
-1.0
0
1.0
2.0
3.0
4.0
5.0
6.0
7.0
8.0
9.0
10.0
11.0
12.0
Y : parts per Million : Proton
12.0
11.0
10.0
9.0
8.0
7.0
6.0
5.0
4.0
3.0
2.0
1.0
0
-1.0
-2.0
X : parts per Million : Proton
0.1
abundance

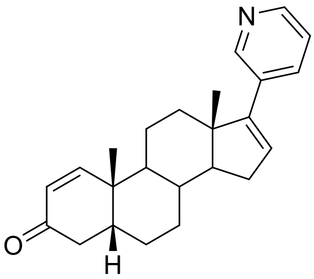

N
O
H

1b
